# Investigating phenotypes of pulmonary COVID-19 recovery: A longitudinal observational prospective multicenter trial

**DOI:** 10.7554/eLife.72500

**Published:** 2022-02-08

**Authors:** Thomas Sonnweber, Piotr Tymoszuk, Sabina Sahanic, Anna Boehm, Alex Pizzini, Anna Luger, Christoph Schwabl, Manfred Nairz, Philipp Grubwieser, Katharina Kurz, Sabine Koppelstätter, Magdalena Aichner, Bernhard Puchner, Alexander Egger, Gregor Hoermann, Ewald Wöll, Günter Weiss, Gerlig Widmann, Ivan Tancevski, Judith Löffler-Ragg

**Affiliations:** 1 Department of Internal Medicine II, Medical University of Innsbruck Innsbruck Austria; 2 Department of Radiology, Medical University of Innsbruck Innsbruck Austria; 3 The Karl Landsteiner Institute Muenster Austria; 4 Central Institute of Medical and Chemical Laboratory Diagnostics, University Hospital Innsbruck Innsbruck Austria; 5 Munich Leukemia Laboratory Munich Germany; 6 Department of Internal Medicine, St. Vinzenz Hospital Zams Austria; Fred Hutchinson Cancer Research Center United States; Radboud University Medical Centre Netherlands

**Keywords:** COVID-19, long COVID, post-COVID-19 syndrome, pulmonary recovery, computed tomography, machine learning, Human

## Abstract

**Background:**

The optimal procedures to prevent, identify, monitor, and treat long-term pulmonary sequelae of COVID-19 are elusive. Here, we characterized the kinetics of respiratory and symptom recovery following COVID-19.

**Methods:**

We conducted a longitudinal, multicenter observational study in ambulatory and hospitalized COVID-19 patients recruited in early 2020 (n = 145). Pulmonary computed tomography (CT) and lung function (LF) readouts, symptom prevalence, and clinical and laboratory parameters were collected during acute COVID-19 and at 60, 100, and 180 days follow-up visits. Recovery kinetics and risk factors were investigated by logistic regression. Classification of clinical features and participants was accomplished by unsupervised and semi-supervised multiparameter clustering and machine learning.

**Results:**

At the 6-month follow-up, 49% of participants reported persistent symptoms. The frequency of structural lung CT abnormalities ranged from 18% in the mild outpatient cases to 76% in the intensive care unit (ICU) convalescents. Prevalence of impaired LF ranged from 14% in the mild outpatient cases to 50% in the ICU survivors. Incomplete radiological lung recovery was associated with increased anti-S1/S2 antibody titer, IL-6, and CRP levels at the early follow-up. We demonstrated that the risk of perturbed pulmonary recovery could be robustly estimated at early follow-up by clustering and machine learning classifiers employing solely non-CT and non-LF parameters.

**Conclusions:**

The severity of acute COVID-19 and protracted systemic inflammation is strongly linked to persistent structural and functional lung abnormality. Automated screening of multiparameter health record data may assist in the prediction of incomplete pulmonary recovery and optimize COVID-19 follow-up management.

**Funding:**

The State of Tyrol (GZ 71934), Boehringer Ingelheim/Investigator initiated study (IIS 1199-0424).

**Clinical trial number:**

ClinicalTrials.gov: NCT04416100

## Introduction

The ongoing COVID-19 pandemic challenges health-care systems. As of December 2021, the John Hopkins dashboard (Dong et al., 2020)⁠ reports 276 million cases and 5.4 million COVID-19-related deaths worldwide (Johns Hopkins Coronavirus Resource Center, 2021)⁠. Although the vast majority of COVID-19 patients display mild disease, approximately 10–15% of cases progress to a severe condition and approximately 5% suffer from critical illness (Perez-Saez, 2021; Huang et al., 2020). Similar to severe acute respiratory syndrome (SARS) (Hui et al., 2005; Ng et al., 2004; Ngai et al., 2010; Lam et al., 2009)⁠, a significant portion of COVID-19 patients report lingering or recurring clinical impairment and cardiopulmonary recovery may take several months to years (Sonnweber et al., 2021; Sahanic et al., 2021; Caruso et al., 2021; Huang et al., 2021b; Huang et al., 2021a; Faverio et al., 2021; Hellemons et al., 2021; Zhou et al., 2021; Venkatesan, 2021)⁠. This observation has led to the introduction of the term ‘long COVID,’ defined by the persistence of COVID-19 symptoms for more than 4 weeks, and the ‘post-acute sequelae of COVID-19’ (PASC) referring to symptom persistence for more than 12 weeks (Sahanic et al., 2021; Shah et al., 2021; Sudre et al., 2021b)⁠. Evidence-based strategies for prediction, monitoring, and treatment of PASC are urgently needed (Raghu and Wilson, 2020)⁠.

We herein prospectively analyzed the prevalence of nonresolving structural and functional lung abnormalities and persistent COVID-19-related symptoms 6 months after diagnosis. Using univariate risk modeling as well as multiparameter clustering and machine learning (ML), we investigated sets of risk factors and tested the operability of ML classifiers at predicting protracted lung and symptom recovery. The classification and prediction procedures were implemented in an open-source risk assessment tool (https://im2-ibk.shinyapps.io/CovILD/).

## Methods

### Study design

The CovILD (‘Development of interstitial lung disease in COVID-19’) multicenter, longitudinal observational study (Sonnweber et al., 2021) was initiated in April 2020. Adult residents of Tyrol, Austria, with symptomatic, PCR-confirmed SARS-CoV-2 infection (WHO, 2021)⁠ were enrolled by the Department of Internal Medicine II at the Medical University of Innsbruck (primary follow-up center), St. Vinzenz Hospital in Zams, and the acute rehabilitation facility in Münster (Table 1). The participants were diagnosed with COVID-19 between 3 March and 29 June 2020. In course of the study, including the 2020 SARS-CoV-2 outbreak and follow-up visits, the regional health system was able to guarantee an unrestricted, optimal standard of diagnostics and care for all participants. Corticosteroids were not standard of care during the recruitment period of the study, thus were not administered as a therapy of acute COVID-19. Some participants with nonresolving pneumonia received systemic steroids beginning from week 4 post diagnosis at the discretion of the physician (Table 2). The analysis endpoints were the presence of any, mild (severity score ≤ 5), and moderate-to-severe (severity score > 5) lung computed tomography (CT) abnormalities, impaired lung function (LF), and persistent COVID-19 symptoms at the 180-day follow-up visit (Table 3).

**Table 1.** Characteristics of the study population.

Characteristics (% cohort)	
Total participants – no.	145
Mean age, years	57.3 (SD = 14.3)
Female sex	42.4% (n = 63)
Obesity (body mass index >30 kg/m^2^)	19.3% (n = 28)
Ex-smoker	39.3% (n = 57)
Active smoker	2.8% (n = 4)
**Acute COVID-19 severity (% cohort**)	
Mild: outpatient	24.8% (n = 36)
Moderate: inpatient without oxygen therapy	25.5% (n = 37)
Severe: inpatient with oxygen therapy	27.6% (n = 40)
Critical: intensive care unit	22.1% (n = 32)
**Comorbidities (% cohort**)	
None	22.8% (n = 33)
Cardiovascular disease	40% (n = 58)
Pulmonary disease	18.6% (n = 27)
Metabolic disease	43.4% (n = 63)
Chronic kidney disease	6.9% (n = 10)
Gastrointestinal tract diseases	13.8% (n = 20)
Malignancy	11.7% (n = 17)

**Table 2.** Hospitalization and medication during acute COVID-19.

Parameter	Outpatient (n = 36)	Hospitalized (n = 37)	Hospitalized oxygen therapy (n = 40)	Hospitalized intensive care unit (n = 32)
Mean hospitalization time, days	0 (SD = 0)	6.9 (SD = 3.6)	11.8 (SD = 6.3)	34.8 (SD = 15.7)
Hospitalized >7 days	0% (n = 0)	43.2% (n = 16)	80% (n = 32)	100% (n = 32)
Anti-infectives	11.1% (n = 4)	45.9% (n = 17)	72.5% (n = 29)	87.5% (n = 28)
Antiplatelet drugs	2.8% (n = 1)	10.8% (n = 4)	22.5% (n = 9)	25% (n = 8)
Anticoagulatives	2.8% (n = 1)	2.7% (n = 1)	5% (n = 2)	15.6% (n = 5)
Corticosteroids*†	2.8% (n = 1)	5.4% (n = 2)	22.5% (n = 9)	40.6% (n = 13)
Immunosuppression‡†	0% (n = 0)	2.7% (n = 1)	5% (n = 2)	9.4% (n = 3)

* From the week 4 post diagnosis on, at the discretion of the physician.

† Subsumed under ‘immunosuppression, acute COVID-19’ for data analysis.

‡ Immunosuppressive medication prior to COVID-19.

**Table 3.** Radiological, functional, and clinical study outcomes.

Outcome	60-day follow-up	100-day follow-up	180-day follow-up
Any lung CT abnormalities (complete: n = 103)	74.8% (n = 77)	60.2% (n = 62)	48.5% (n = 50)
Mild lung CT abnormalities (severity score ≤ 5) (complete: n = 103)	26.2% (n = 27)	36.9% (n = 38)	29.1% (n = 30)
Moderate-to-severe CT abnormalities (severity score > 5) (complete: n = 103)	48.5% (n = 50)	23.3% (n = 24)	19.4% (n = 20)
Functional lung impairment (complete: n = 116)	39.7% (n = 46)	37.1% (n = 43)	33.6% (n = 39)
Persistent symptoms (complete: n = 145)	79.3% (n = 115)	67.6% (n = 98)	49% (n = 71)

CT = computed tomography.

**Figure 1.**
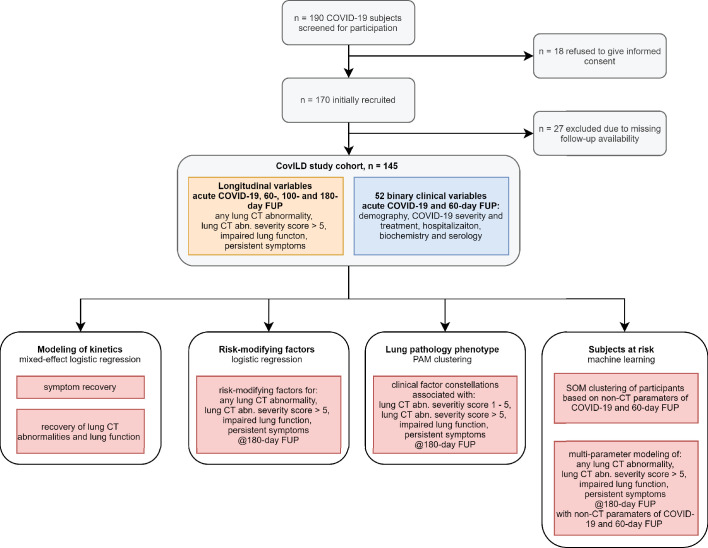
Study inclusion flow diagram and analysis scheme.

In total, 190 COVID-19 patients were screened for participation. Thereof, n = 18 subjects refused to give informed consent, n = 27 declared difficulties to appear at the study follow-ups. Data of n = 145 participants were eligible for analysis (Figure 1). All participants gave written informed consent. The study was approved by the Institutional Review Board at the Medical University of Innsbruck (approval number: 1103/2020) and registered at ClinicalTrials.gov (NCT04416100).

### Procedures

We retrospectively assessed patient characteristics during acute COVID-19 and performed follow-up investigations at 60 days (63 ± 23 days [mean ± SD]; visit 1), 100 days (103 ± 21 days; visit 2), and 180 days (190 ± 15 days; visit 3) after diagnosis of COVID-19. Each visit included symptom and physical performance assessment with a standardized questionnaire, LF testing, standard laboratory testing, and a CT scan of the chest. The variables available for analysis with their stratification schemes are listed in Appendix 1—table 1.

Serological markers were determined in certified laboratories (Central Institute of Clinical and Chemical Laboratory Diagnostics, Rheumatology and Infectious Diseases Laboratory, both at the University Hospital of Innsbruck). C-reactive protein (CRP), interleukin-6 (IL-6), N-terminal pro natriuretic peptide (NT-proBNP), and serum ferritin were measured using a Roche Cobas 8000 analyzer. D-dimer was determined with a Siemens BCS-XP instrument using the Siemens D-Dimer Innovance reagent. Anti-S1/S2 protein SARS-CoV-2 immunoglobulin gamma (IgG) were quantified with LIAISON chemoluminescence assay (DiaSorin, Italy), expressed as binding antibody units (BAU, conversion factor = 5.7) and stratified by quartiles (Ferrari et al., 2021)⁠.

Low-dose (100 kVp tube potential) craniocaudal CT scans of the chest were acquired without iodine contrast and without ECG gating on a 128-slice multidetector CT (128 × 0.6 mm collimation, 1.1 spiral pitch factor, SOMATOM Definition Flash, Siemens Healthineers, Erlangen, Germany). In case of clinically suspected pulmonary embolism, CT scans were performed with a contrast agent. Axial reconstructions were done with 1 mm slices. CT scans were evaluated for ground-glass opacities, consolidations, bronchial dilation, and reticulations as defined by the Fleischner Society. Lung findings were graded with a semi-quantitative CT severity score (0–25 points) (Sonnweber et al., 2021)⁠.

Impaired LF was defined as (1) forced vital capacity (FVC) < 80% or (2) forced expiratory volume in 1 s (FEV_1_) < 80%, or (3) FEV_1_:FVC < 70% or (4) total lung capacity (TLC) < 80% or (5) diffusing capacity of carbon monoxide (DLCO) < 80% predicted.

### Statistical analysis

Statistical analyses were performed with R version 4.0.5 (Figure 1). Data transformation and visualization were accomplished by *tidyverse* (Wickham et al., 2019)⁠, *ggplot2* (Wickham, 2016)⁠, *ggvenn*, *plotROC* (Sachs, 2017),⁠ and *cowplot* (Wilke, 2019)⁠ packages. The recorded variables were binarized as shown in Appendix 1—table 1. Acute COVID-19 severity strata were defined as presented in Table 1. p-Values were corrected for multiple comparisons with the Benjamini–Hochberg method (Benjamini and Hochberg, 1995), and effects were termed significant for p<0.05.

### Variable overlap, kinetics, and risk modeling

Overlap between the 180-day follow-up outcome features was assessed by analysis of quasi-proportional Venn plots (package *nVennR*) (Pérez-Silva et al., 2018)⁠ and calculation of the Cohen’s κ statistic (package *vcd*) (Fleiss et al., 1969)⁠. Kinetics of binary outcome variables in participants subsets with the complete longitudinal data record was modeled with mixed-effect logistic regression (random effect: individual, fixed effect: time, packages *lme4* [Bates et al., 2015]⁠ and *lmerTest* [Kuznetsova et al., 2017]⁠). Analyses in the severity groups were done with separate models. Significance was assessed by the likelihood ratio test (LRT) against the random-term-only model. Univariate risk modeling was performed with fixed-effect logistic regression (Appendix 1—table 2). Odds ratio (OR) significance was determined by Wald Z test. In-house-developed linear modeling wrappers around base R tools are available at https://github.com/PiotrTymoszuk/lmqc.

### Cluster analysis

Clustering of non-CT and non-LF binary clinical features (Appendix 1—table 1) was accomplished with PAM algorithm (partitioning around medoids, package *cluster*) (Amato et al., 2019)⁠ and simple matching distance (SMD, package *nomclust*) (Boriah et al., 2008)⁠. Association analysis for the participants was performed with a combined procedure involving clustering of the observations by the self-organizing map algorithm (SOM, 4 × 4 hexagonal grid, SMD distance, *kohonen* package), followed by clustering of the SOM nodes by the Ward.D2 hierarchical clustering algorithm (Euclidean distance, *hclust*() function, package *stats*) (Vesanto and Alhoniemi, 2000; Kohonen, 1995; Wehrens and Kruisselbrink, 2018)⁠. Clustering analyses were performed in the participant subset with the complete set of clustering variables. The selection of the optimal clustering algorithm was motivated by the highest ratio of between-cluster to total variance and the best stability measured by mean classification error in 20-fold cross-validation (CV) (Figure 6—figure supplement 1A and B, Figure 7—figure supplement 1A and B; Lange et al., 2004)⁠. The optimal cluster number was determined by the bend of the within-cluster sum-of-squares curve (function *fviz_nbclust*(), package *factoextra*) and by the stability in 20-fold CV (Figure 6—figure supplement 1C and D, Figure 7—figure supplement 1D and F; Lange et al., 2004; Wang, 2010)⁠, as well as by a visual inspection of the SOM node clustering dendrograms (Figure 7—figure supplement 1E). Assignment of 180-day follow-up outcome features to the clusters of clinical parameters was accomplished with a k-nearest neighbor (kNN) label propagation algorithm (Appendix 1—table 3; Sahanic et al., 2021; Leng et al., 2013)⁠. Cluster assignment visualization in a four-dimensional principal analysis score plot was done with the *PCAproj*() tool (package *pcaPP*) (Croux et al., 2007)⁠. To determine the importance of particular clustering variables, the variance (between-cluster to total variance ratio) between the initial cluster structure and the structure with random resampling of the variable was compared, as initially proposed for the random forests ML classifier (Breiman, 2001)⁠. Frequencies of the outcome events in the participant clusters were compared with χ^2^ test. In-house-developed association analysis wrappers are available at https://github.com/PiotrTymoszuk/clustering-tools-2.

### Machine learning

ML classifiers C5.0 (package *C50*) (Quinlan, 1993)⁠, random forests (*randomForest*) (Breiman, 2001)⁠, support vector machines with radial kernel (*kernlab*) (Weston and Watkins, 1998)⁠, neural networks (*nnet*) (Ripley, 2014)⁠, and elastic net (*glmnet*) (Friedman et al., 2010)⁠ were trained to predict the 180-day follow-up outcomes employing non-CT and non-LF binary explanatory features (Appendix 1—table 1). The ML training was performed in the participant subsets with the complete set of explanatory and outcome variables. The training, optimization, and CV (20-fold, five repetitions) were accomplished by the *train*() tool from *caret* package, with the Cohen’s κ statistic as a model selection metric (Appendix 1—table 4; Kuhn, 2008)⁠. Classifier ensembles were constructed with the elastic net procedure (*caretStack*() function, *caretEnsemble* package, Appendix 1—table 4; Deane-Mayer and Knowles, 2019)⁠. Classifier performance in the training cohort and CV was assessed by receiver-operating characteristics (ROCs), Cohen’s κ and accuracy (packages *caret* and *vcd,* Appendix 1—table 5; Fleiss et al., 1969; Kuhn, 2008)**⁠**. Variable importance measures were extracted from the C5.0 (percent variable usage, *c5imp*() function, package *C50*) (Quinlan, 1993)⁠, random forests (Δ Gini index, *importance*(), package *randomForest*) (Breiman, 2001)⁠, and elastic net classifiers (regression coefficient β, *coef*(), package *glmnet*) (Friedman et al., 2010)⁠.

### Pulmonary recovery assessment app

Participant clustering and ML classifiers trained in the CovILD cohort were implemented in an open-source online pulmonary assessment R shiny app (https://im2-ibk.shinyapps.io/CovILD/; code: https://github.com/PiotrTymoszuk/COVILD-recovery-assessment-app). Prediction of the cluster assignment based on the user-provided patient data is done by the kNN label propagation algorithm (Sahanic et al., 2021; Leng et al., 2013)⁠.

## Results

### Patient characteristics

The CovILD study participants (n = 145) were predominantly male (57.8%), age ranging between 19 and 87 years. 77.2% of participants displayed preexisting comorbidity, predominantly cardiovascular and metabolic disease. The cohort included mild (outpatient care, 24.8%), moderate (hospitalization without oxygen supply, 25.5%), severe (hospitalization with oxygen supply, 27.6%), and critical (intensive care unit [ICU] treatment, 22.1%) cases of acute COVID-19 (Table 1). The majority of hospitalized participants received anti-infectives during acute COVID-19, anticoagulative, and/or antiplatelet treatment introduced primarily in the ventilated patients. Systemic steroid administration was initiated at the discretion of the physician beginning from week 4 after diagnosis (Table 2).

### Clinical recovery after COVID-19

**Figure 2.**
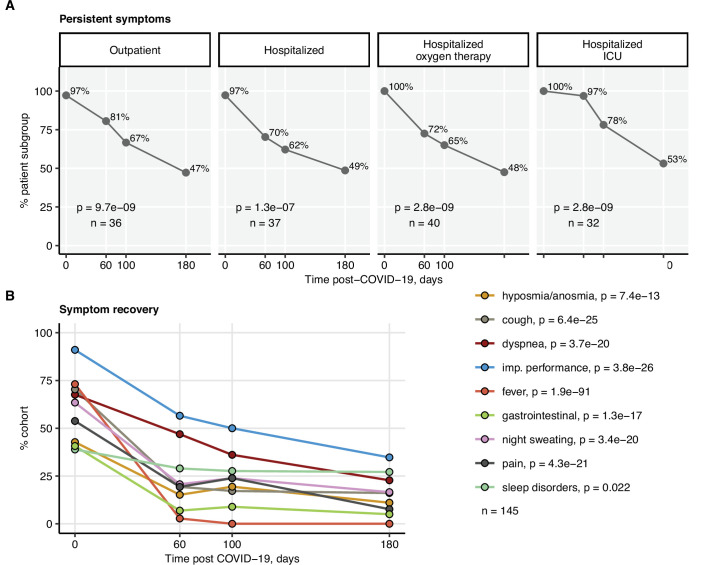
Kinetic of recovery from COVID-19 symptoms. Recovery from any COVID-19 symptoms was investigated by mixed-effect logistic modeling (random effect: individual; fixed effect: time). Significance was determined by the likelihood ratio test corrected for multiple testing with the Benjamini–Hochberg method, and p-values and the numbers of complete observations are indicated in the plots. (**A**) Frequencies of individuals with any symptoms in the study cohort stratified by acute COVID-19 severity. (**B**) Frequencies of participants with particular symptoms. imp.: impaired.

Most patients, irrespective of the acute COVID-19 severity, showed a significant resolution of disease symptoms over time (Figure 1, Figure 2A). Persistent complaints at the 6-month follow-up were reported by 49% of the study subjects (Table 3), with self-reported impaired physical performance (34.7%), sleep disorders (27.1%), and exertional dyspnea (22.8%) as leading manifestations. The frequency of all investigated symptoms declined significantly, even though the pace of their resolution was remarkably slower in the late (100- and 180-day follow-ups) than in the early recovery phase (acute COVID-19 till 60-day follow-up) (Figure 2B).

**Figure 3.**
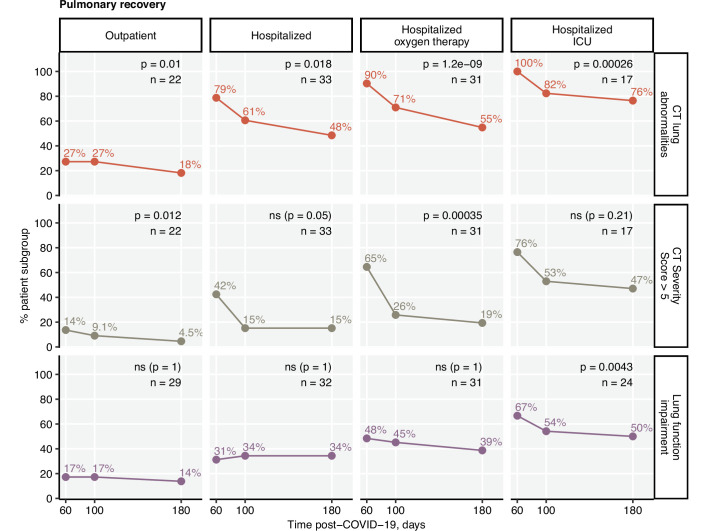
Kinetic of pulmonary recovery. Recovery from any lung computed tomography (CT) abnormalities, moderate-to-severe lung CT abnormalities (severity score > 5), and recovery from functional lung impairment were investigated in the participants stratified by acute COVID-19 severity by mixed-effect logistic modeling (random effect: individual; fixed effect: time). Significance was determined by the likelihood ratio test corrected for multiple testing with the Benjamini–Hochberg method. Frequencies of the given abnormality at the indicated time points are presented, and p-values and the numbers of complete observations are indicated in the plots.

Impaired LF was observed in 33.6% of the participants at the 6-month follow-up (Table 3). Except for the critical COVID-19 survivors (60 days: 66.7%; 180 days post-COVID-19: 50%), no significant reduction in the frequency of LF impairment over time was observed (Figure 3). At the 6-month follow-up, structural lung abnormalities were found in 48.5% of patients and moderate-to-severe radiological lung alterations (CT severity score > 5) were present in 19.4% of participants (Table 3). The majority of the participants with impaired LF displayed radiological lung findings. However, a substantial fraction of CT abnormalities, especially mild ones, were accompanied neither by persistent symptoms nor by LF deficits (Figure 3—figure supplement 1, Figure 3—figure supplement 2**,** Figure 3—figure supplement 3A).

**Figure 3—figure supplement 1.**
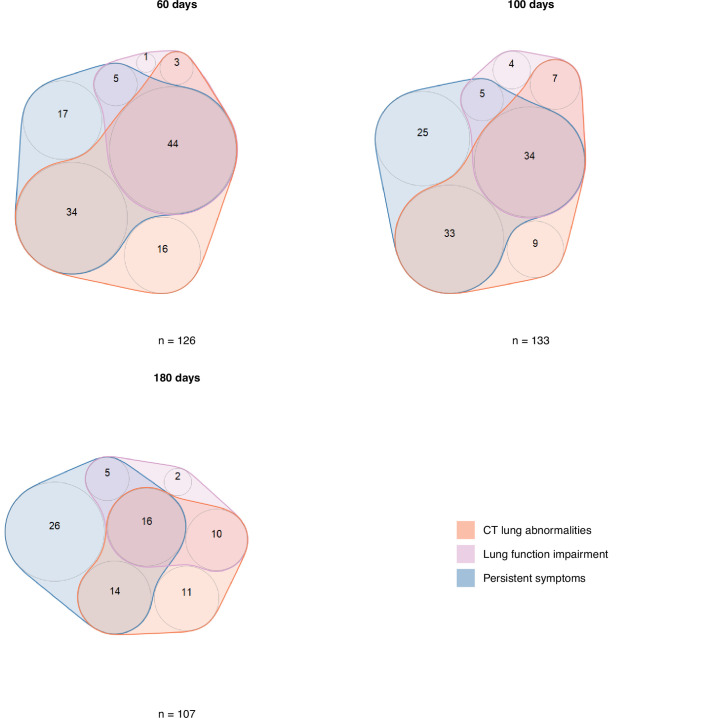
Co-occurrence of lung computed tomography (CT) abnormalities, functional lung impairment, and any persistent symptoms. Numbers and percentages of the study participants with any persistent symptoms, functional lung impairment, or lung CT abnormalities at the consecutive follow-up visits presented in quasi-proportional Venn diagrams. The numbers of participants with CT abnormalities, lung function (LF) impairment, and persistent symptoms are indicated in the diagrams, and the numbers of complete observations are shown under the plots.

**Figure 3—figure supplement 2.**
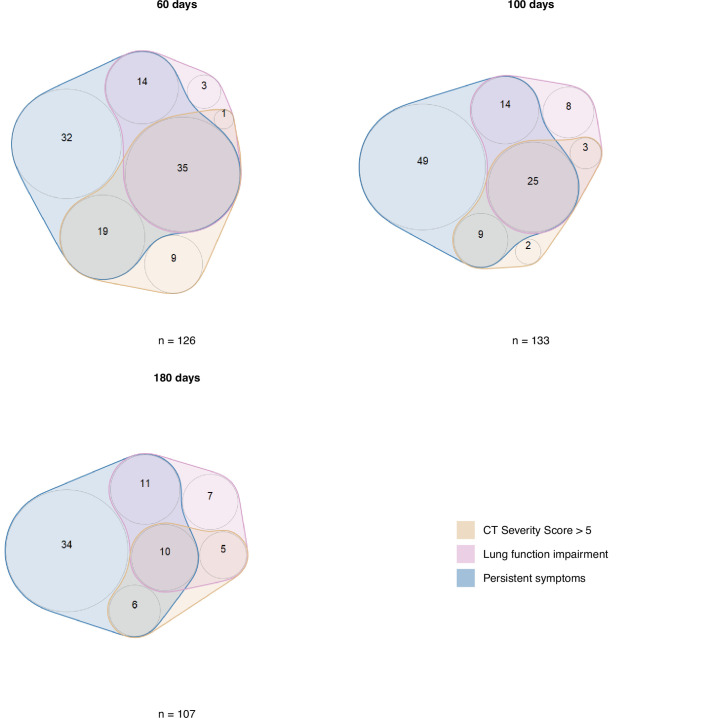
Co-occurrence of moderate-to-severe lung computed tomography (CT) abnormalities, functional lung impairment, and any persistent symptoms. Numbers and percentages of the study participants with any persistent symptoms, functional lung impairment, or moderate-to-severe lung CT abnormalities (severity score > 5) at the consecutive follow-up visits presented in quasi-proportional Venn diagrams. The numbers of participants with CT abnormalities, lung function (LF) impairment, and persistent symptoms are indicated in the diagrams, and the numbers of complete observations are shown under the plots.

**Figure 3—figure supplement 3.**
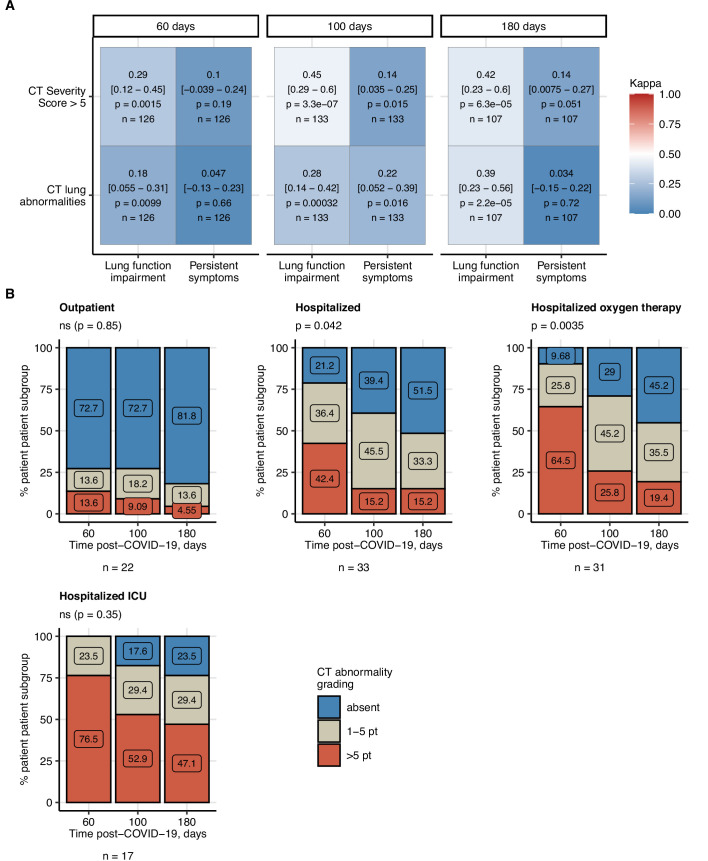
Frequency of mild and moderate-to-severe lung computed tomography (CT) abnormalities. Prognostic value of functional lung impairment and persistent symptoms for prediction of radiological lung abnormalities. (**A**) Relevance of functional lung impairment and persistent COVID-19 symptoms at predicting any lung CT abnormalities and moderate-to-severe lung CT abnormalities (severity score > 5) at the consecutive follow-up visits. The concordance of the outcome variables was determined by Cohen’s κ coefficient. Statistical significance (κ / = 0) was assessed by two-tailed *t*-test corrected for multiple testing with the Benjamini–Hochberg method. Kappa with 95% confidence intervals and p values are presented as a heat map. The number of complete observations is indicated in the plot. (**B**) Percentages of mild (severity score ≤ 5) and moderate-to-severe lung CT abnormalities at the consecutive follow-up visits in the study participants stratified by the severity of acute COVID-19. Statistical significance of frequency differences was determined by χ^2^ test for trend corrected for multiple testing with the Benjamini–Hochberg method. The number of complete observations is indicated under the plots.

The frequency, scoring, and recovery of CT lung findings were related to the severity of acute infection. Pulmonary lesions scored > 5 CT severity points at the 180-day follow-up were most frequent in the individuals with severe and critical acute COVID-19 (Figure 3—figure supplement 3). Notably, the hospitalized group with oxygen therapy demonstrated the fastest recovery kinetics. As for the symptom resolution, LF and CT lung recovery decelerated in the late phase of COVID-19 convalescence (Figure 3).

### Risk factors of protracted recovery

**Figure 4.**
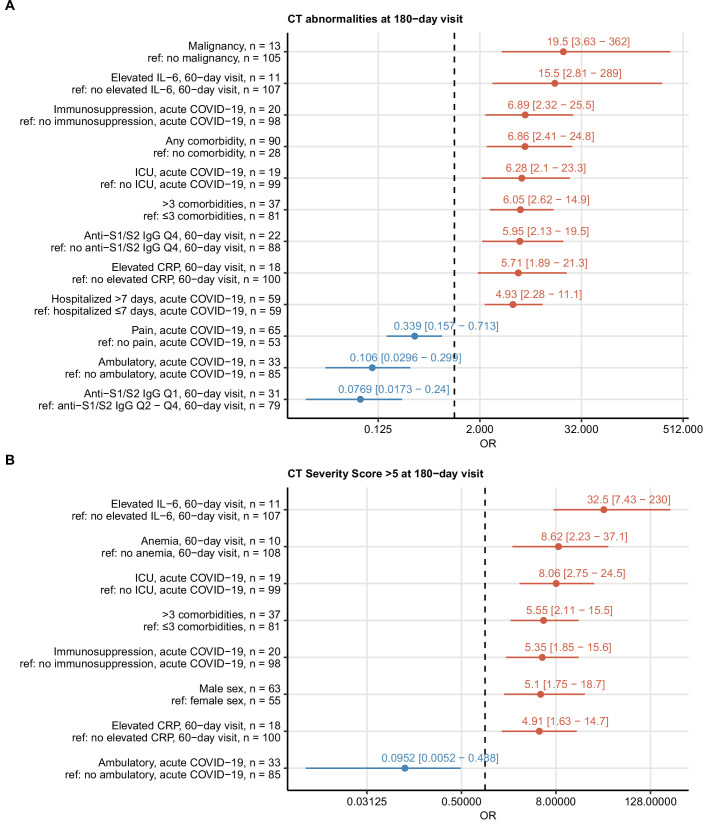
Risk factors of persistent radiological lung abnormalities. Association of 52 binary explanatory variables (Appendix 1—table 1) with the presence of any lung computed tomography (CT) abnormalities (**A**) or moderate-to-severe lung CT abnormalities (severity score > 5) (**B**) at the 180-day follow-up visit was investigated with a series of univariate logistic models (Appendix 1—table 2). Odds ratio (OR) significance was determined by Wald Z test and corrected for multiple testing with the Benjamini–Hochberg method. ORs with 95% confidence intervals for significant favorable and unfavorable factors are presented in forest plots. Model baseline (ref) and numbers of complete observations are presented in the plot axis text. Q1, Q2, Q3, Q4: first, second, third, and fourth quartile of anti-S1/S2 IgG titer; ICU: intensive care unit.

**Figure 5.**
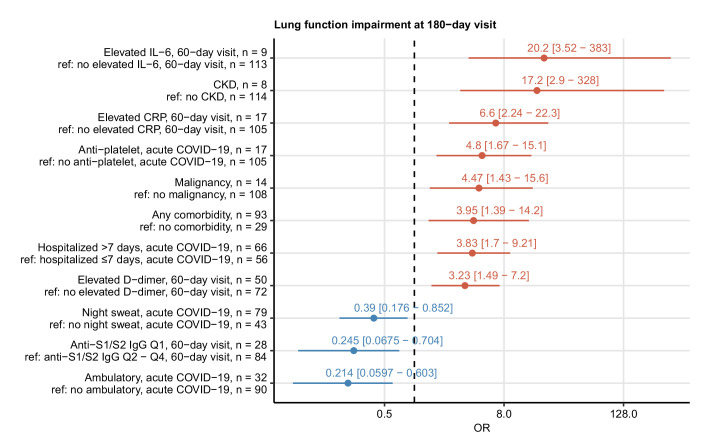
Risk factors of persistent functional lung impairment. Association of 52 binary explanatory variables (Appendix 1—table 1) with the presence of functional lung impairment at the 180-day follow-up visit was investigated with a series of univariate logistic models (Appendix 1—table 2). Odds ratio (OR) significance was determined by Wald Z test and corrected for multiple testing with the Benjamini–Hochberg method. ORs with 95% confidence intervals for the significant favorable and unfavorable factors are presented in a forest plot. Model baseline (ref) and n numbers of complete observations are presented in the plot axis text. Q1, Q2, Q3, Q4: first, second, third, and fourth quartile of anti-S1/S2 IgG titer; CKD: chronic kidney disease.

To identify risk factors of delayed recovery at the 6-month follow-up, we screened a set of 52 binary clinical parameters (Appendix 1—table 1) recorded during acute COVID-19 and at the 60-day visit by univariate modeling (Appendix 1—table 2). By this means, no significant correlates for long-term symptom persistence were identified. Risk factors and readouts of severe and critical COVID-19 including multimorbidity, malignancy, male sex, prolonged hospitalization, ICU stay, and immunosuppressive therapy were significantly associated with persistent CT (Figure 4) and LF abnormalities (Figure 5). Persistently elevated inflammatory markers, IL-6 (>7 ng/L) and CRP (>0.5 mg/L), were strong unfavorable risk factors for incomplete radiological and functional pulmonary recovery. Additionally, the biochemical readout of microvascular inflammation, D-dimer (>500 pg/mL) was significantly linked to LF deficits. Low serum anti-S1/S2 IgG titers at the 60-day follow-up and ambulatory acute COVID-19 correlated with an improved pulmonary recovery (Figures 4 and 5).

### Clusters of clinical features linked to persistent symptoms and lung abnormalities

Employing the unsupervised PAM algorithm (Amato et al., 2019)⁠, three clusters of co-occurring non-CT and non-LF clinical features of acute COVID-19 and early convalescence (Appendix 1—table 1) were identified (Figure 6—figure supplement 1, Appendix 1—table 3): (1) cluster 1 with male sex, hypertension, and cardiovascular and metabolic comorbidity; (2) cluster 2, including characteristics of acute COVID-19 severity and inflammatory markers; and (3) cluster 3 consisting of acute and persistent COVID-19 symptoms (Figure 6—figure supplement 2, Appendix 1—table 3).

**Figure 6.**
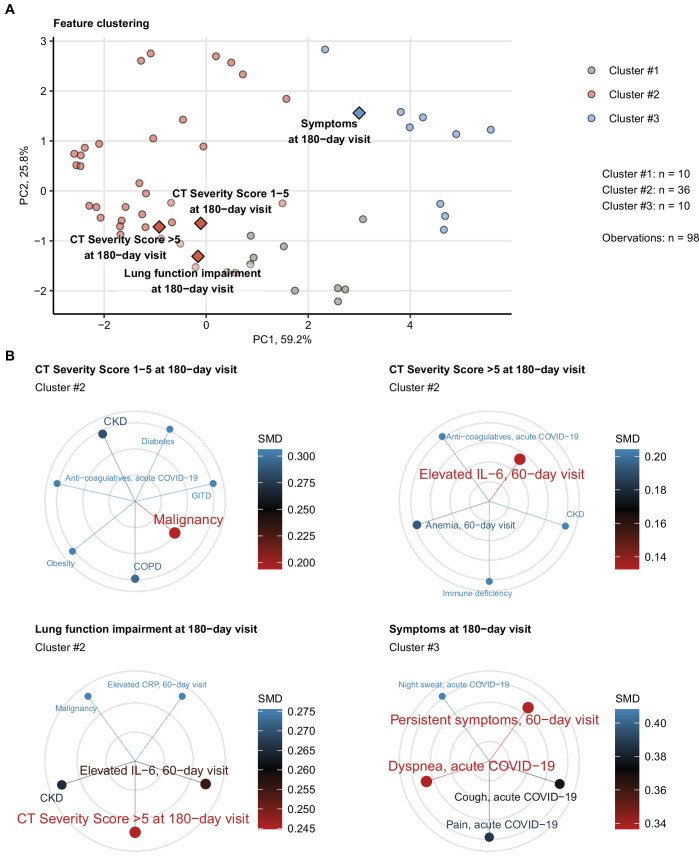
Association of incomplete symptom, lung function, and radiological lung recovery with demographic and clinical parameters of acute COVID-19 and early recovery. Clustering of 52 non-computed tomography (non-CT) and non-lung function binary explanatory variables recorded for acute COVID-19 or at the early 60-day follow-up visit (Appendix 1—table 1) was investigated by partitioning around medoids (PAM) algorithm with simple matching distance (SMD) dissimilarity measure (Figure 6—figure supplement 1, Appendix 1—table 3). The cluster assignment for the outcome variables at the 180-day follow-up visit (persistent symptoms, functional lung impairment, mild lung CT abnormalities [severity score ≤ 5] and moderate-to-severe lung CT abnormalities [severity score > 5]) was predicted by k-nearest neighbor (kNN) label propagation procedure. Numbers of complete observations and numbers of features in the clusters are indicated in (**A**). (**A**) Cluster assignment of the outcome variables (diamonds) presented in the plot of principal component (PC) scores. The first two major PCs are displayed. The explanatory variables are visualized as points. Percentages of the data set variance associated with the PC are presented in the plot axes. (**B**) Five nearest neighbors (lowest SMD) of the outcome variables presented in radial plots. Font size, point radius, and color code for SMD values. Q1, Q2, Q3, Q4: first, second, third, and fourth quartile of anti-S1/S2 IgG titer; GITD: gastrointestinal disease; CKD: chronic kidney disease; ICU: intensive care unit; COPD: chronic obstructive pulmonary disease.

The 6-month follow-up outcome variables were incorporated in the cluster structure using kNN prediction (Leng et al., 2013)⁠. Long-term symptom persistence was associated with acute and long-lasting COVID-19 symptoms in cluster 3, whereas pulmonary outcome parameters were grouped with cluster 2 features (Figure 6A, Figure 6—figure supplement 2, Appendix 1—table 3). Preexisting comorbidities such as malignancy, kidney, lung and gastrointestinal disease, obesity, and diabetes were found the closest cluster neighbors of mild CT abnormalities (severity score ≤ 5). Moderate-to-severe structural alterations (severity score > 5) and LF deficits were, in turn, tightly linked to markers of protracted systemic inflammation (IL-6, CRP, anemia of inflammation) (Sonnweber et al., 2020;⁠ Figure 6B).

**Figure 6—figure supplement 1.**
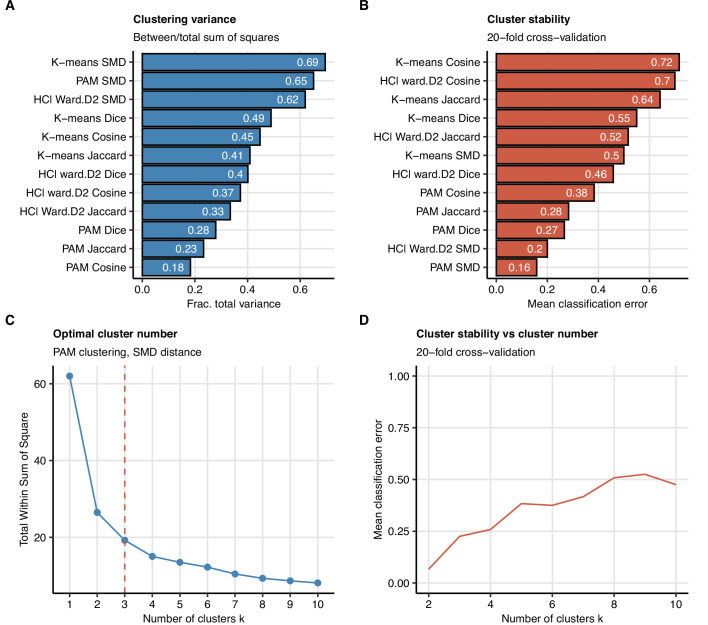
Study feature clustering algorithm. Clustering of 52 non-computed tomography (non-CT) and non-lung function binary explanatory variables recorded for acute COVID-19 or at the early 60-day follow-up visit (Appendix 1—table 1). (**A, B**) Comparison of ‘explained’ variances (between-cluster to total sum-of-squares ratio) (**A**) and cluster stability (mean classification error in 20-fold cross-validation) (**B**) in clustering of the data set with several algorithms with k = 3 centers/branches (algorithms: K-means; PAM: partitioning around medoids; HCl Ward.D2: hierarchical clustering with Ward.D2 method; distances: SMD: simple matching distance; Jaccard, Dice, and Cosine). (**C, D**) The optimal number of the feature clusters in clustering with the optimally performing PAM algorithm with SMD dissimilarity measure was determined by the bend of the total within-cluster sum-of-squares curve (**C**) and confirmed by good stability (low mean classification error) in 20-fold cross-validation (**D**).

**Figure 6—figure supplement 2.**
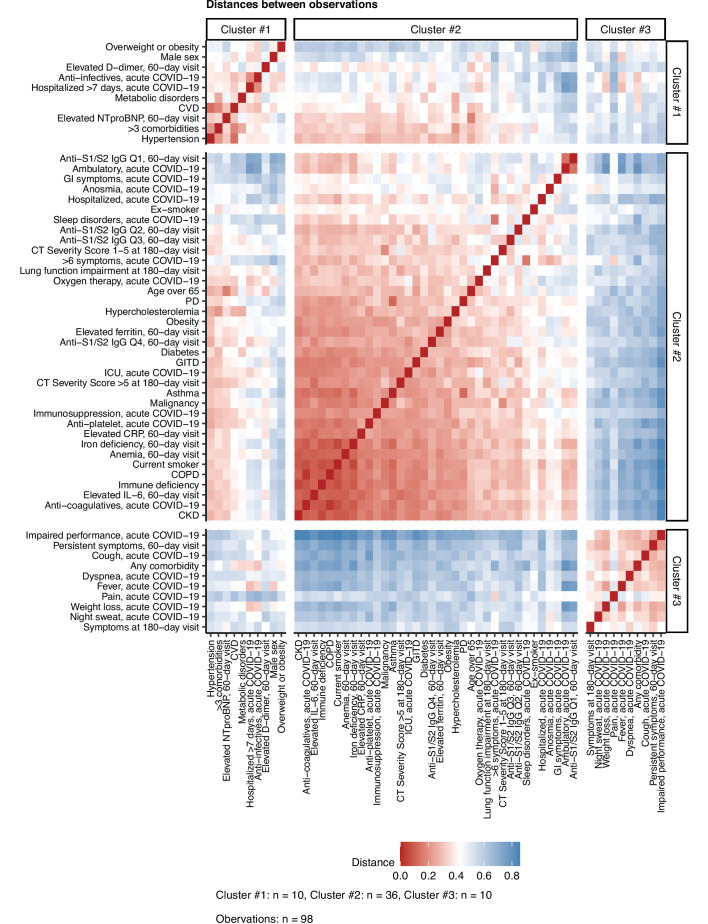
Semi-supervised clustering of mild and moderate-to-severe lung computed tomography (CT) abnormalities, functional lung impairment, and persistent symptoms at the 180-day follow-up with parameters of acute COVID-19 and early convalescence. Clusters of 52 non-CT and non-lung function binary explanatory variables recorded for acute COVID-19 or at the 60-day follow-up visit (Appendix 1—table 1) were defined by the optimally performing partitioning around medoids (PAM) algorithm and simple matching distance (SMD) dissimilarity measure (Figure 6A, Figure 6—figure supplement 1, Appendix 1—table 3). The cluster assignment for the outcome variables at the 180-day follow-up visit (persistent symptoms, functional lung impairment, mild lung CT abnormalities [severity score ≤ 5], and moderate-to-severe lung CT abnormalities [severity score > 5]) was predicted by k-nearest neighbor (kNN) label propagation procedure. SMD between the features and their cluster assignments are shown in a heat map. The numbers of features in the clusters and the total number of observations are indicated under the plot. CVD: cardiovascular disease; Q1, Q2, Q3, Q4: first, second, third, and fourth quartile of anti-S1/S2 IgG titer; GI: gastrointestinal; PD: pulmonary disease; GITD: gastrointestinal disease; ICU: intensive care unit; COPD: chronic obstructive pulmonary disease; CKD: chronic kidney disease.

### Risk stratification for perturbed pulmonary recovery by unsupervised clustering

**Figure 7.**
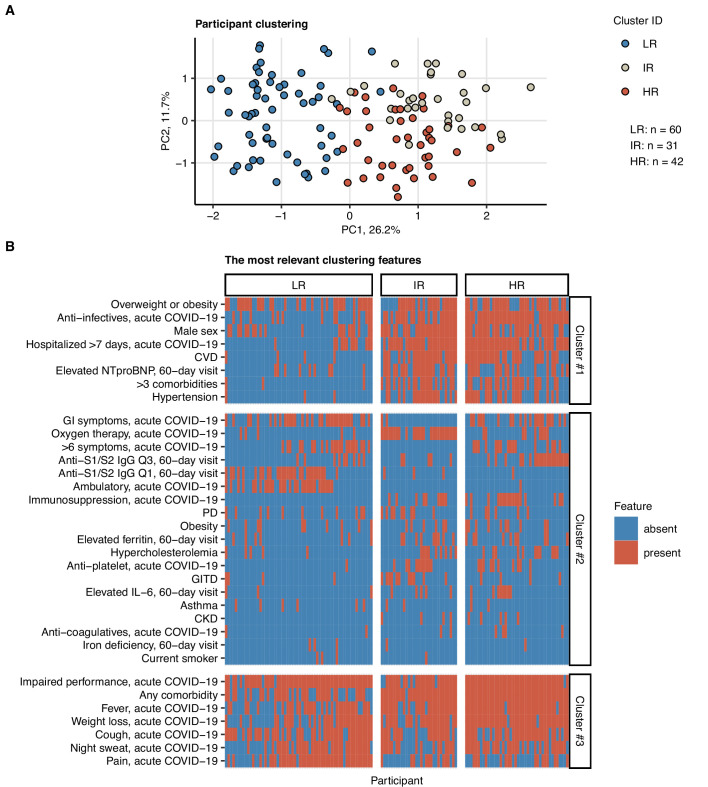
Clustering of the study participants by non-lung function and non-computed tomography (non-CT) clinical features. Study participants (n = 133 with the complete variable set) were clustered with respect to 52 non-CT and non-lung function binary explanatory variables recorded for acute COVID-19 or at the 60-day follow-up visit (Appendix 1—table 1) using a combined self-organizing map (SOM: simple matching distance) and hierarchical clustering (Ward.D2 method, Euclidean distance) procedure (Figure 7—figure supplement 1). The numbers of participants assigned to low-risk (LR), intermediate-risk (IR), and high-risk (HR) clusters are indicated in (**A**). (**A**) Cluster assignment of the study participants in the plot of principal component (PC) scores. The first two major PCs are displayed. Percentages of the data set variance associated with the PC are presented in the plot axes. (**B**) Presence of the most influential clustering features (Figure 7—figure supplement 2) in the participant clusters presented as a heat map. Cluster #1, #2, and #3 refer to the feature clusters defined in Figure 6. Q1, Q2, Q3, Q4: first, second, third, and fourth quartile of anti-S1/S2 IgG titer; GITD: gastrointestinal disease; CKD: chronic kidney disease; CVD: cardiovascular disease; GI: gastrointestinal; PD: pulmonary disease.

**Figure 8.**
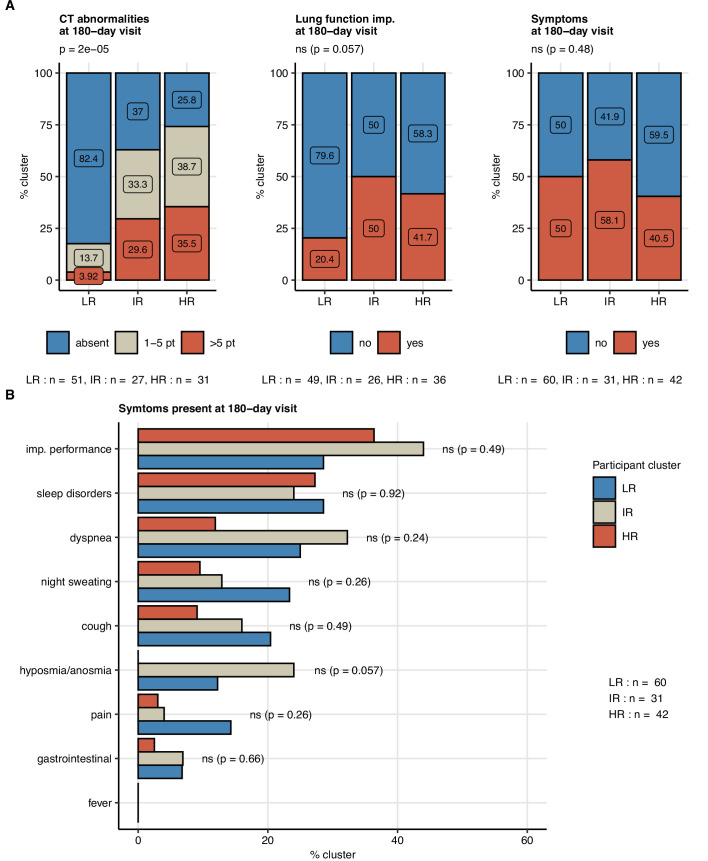
Frequency of persistent radiological lung abnormalities, functional lung impairment, and symptoms in the participant clusters. The clusters of study participants were defined by non-lung function and non-computed tomography (non-CT) features as presented in Figure 7. Frequencies of outcome variables at the 180-day follow-up visit (mild [severity score ≤ 5], moderate-to-severe lung CT abnormalities [severity score > 5], functional lung impairment, and persistent symptoms) were compared between the low-risk (LR), intermediate-risk (IR), and high-risk (HR) participant clusters by χ^2^ test corrected for multiple testing with the Benjamini–Hochberg method. p-Values and numbers of participants assigned to the clusters are indicated in the plots. (**A**) Frequencies of the outcome features in the participant clusters. (**B**) Frequencies of specific symptoms in the participant clusters.

Next, we tested whether subsets of patients at risk of an incomplete 6-month recovery may be defined by a similar clustering procedure employing exclusively non-CT and non-LF clinical variables (Appendix 1—table 1). Applying a combined SOM – hierarchical clustering approach, three clusters of the study participants were identified (Figure 7, Figure 7—figure supplement 1; Vesanto and Alhoniemi, 2000; Kohonen, 1995)⁠. Prolonged hospitalization, anti-infective therapy, overweight or obesity, pain during acute COVID-19, and low anti-S1/S2 titers at the 60-day follow-up were found the most influential clustering features (Figure 7—figure supplement 2; Breiman, 2001)⁠. The patient subsets identified by the SOM approach differed significantly in frequency of radiological lung abnormalities and substantially, yet not significantly, in the frequency of LF impairment at the 180-day follow-up. In particular, most of the individuals assigned to the largest, low-risk (LR) subset were CT and LF abnormality-free. The frequency and severity of radiological pulmonary findings were elevated in the smallest intermediate-risk subset (IR) and peaked in the high-risk (HR) group (Figure 8A). Despite a comparable frequency of long-term symptoms between the LR, IR, and HR subsets (Figure 8A), the HR collective showed the lowest prevalence of dyspnea, cough, night sweating, pain, gastrointestinal manifestations, and complete absence of hyposmia at the 180-day follow-up (Figure 8B). Although the LR subset primarily comprised mild COVID-19 cases and the HR subset ICU survivors, the cluster assignment (IR vs. LR, HR vs. LR) remained an independent correlate of persistent CT and LF abnormalities after adjustment for the acute COVID-19 severity (Figure 8—figure supplement 1).

**Figure 7—figure supplement 1.**
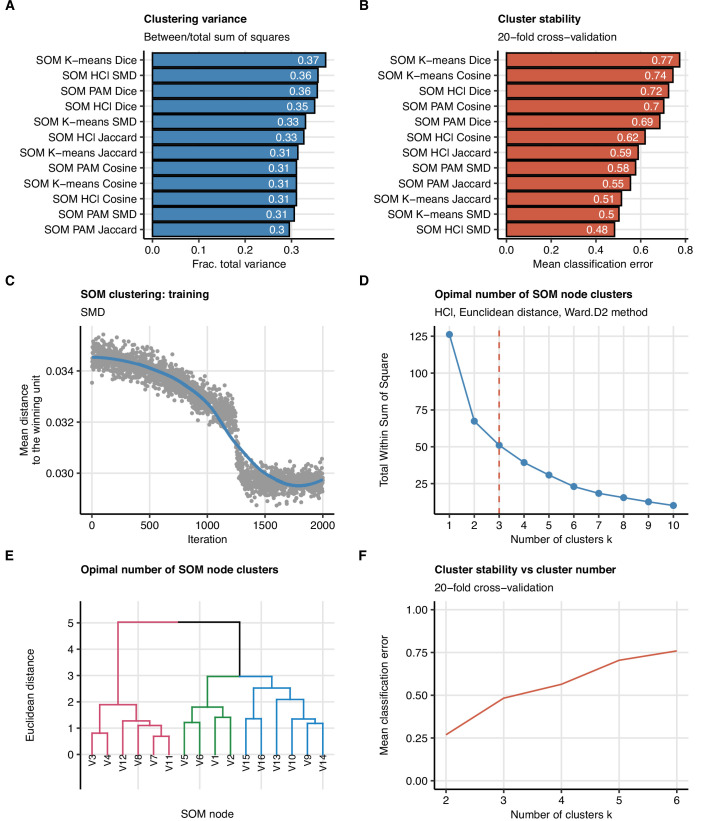
Study participant clustering algorithm. Clustering of the study participants (n = 133 with the complete variable set) with respect to 52 non-computed tomography (non-CT) and non-lung function binary explanatory variables recorded for acute COVID-19 or at the 60-day follow-up visit (Appendix 1—table 1). The procedure involved clustering of the observations with self-organizing maps (SOM, 4 × 4 hexagonal grid, distances: SMD: simple matching distance, Jaccard, Dice, or Cosine) followed by clustering of the SOM nodes (algorithms: HCl ward.D2: hierarchical clustering with Ward.D2 method; K-means; PAM: partitioning around medoids; distance: Euclidean). Different combinations of observation dissimilarity measures and SOM node clustering algorithms were tested in the search for the optimal clustering algorithm. (**A, B**) Comparison of ‘explained’ variances (between-cluster to total sum-of-squares ratio) (**A**) and cluster stability (mean classification error in 20-fold cross-validation) (**B**) in clustering of the data set with different observation distance measures and SOM node clustering algorithms. (C) Training of the SOM algorithm, mean distance to the winning un as a function of lgorithm iterations is presented. Note the mean distance plateau indicative of the algorithm convergence (**D–F**) The optimal number of the SOM node clusters in clustering with the optimally performing SOM HCl algorithm with SMD observation dissimilarity measure. The optimal cluster number was determined by the bend of the total within-cluster sum-of-squares curve (D) and confirmed by visual inspection of the HCl dendrogram (**E**) and good stability (low mean classification error) in 20-fold cross-validation (F).

**Figure 7—figure supplement 2.**
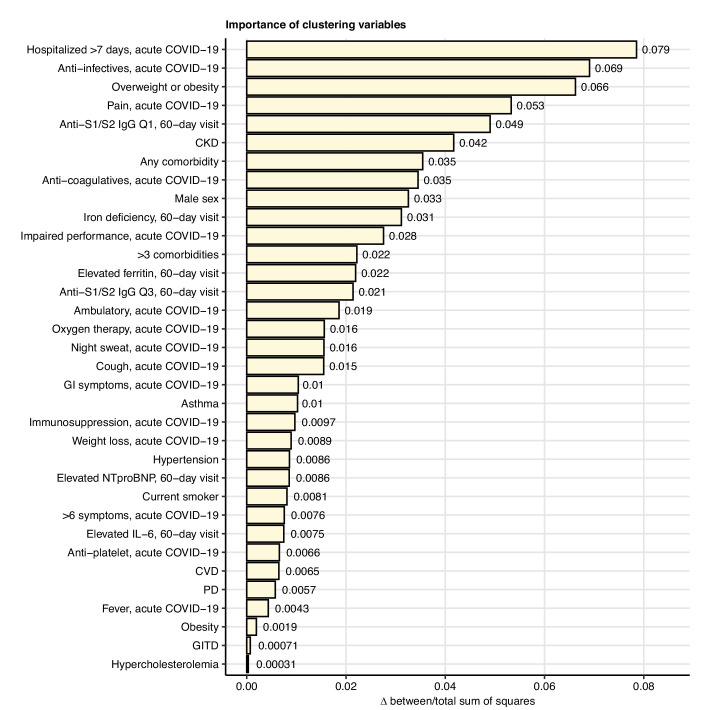
Impact of specific variables on the quality of participant clustering. The clusters of participants clusters were defined with the optimally performing self-organizing map (SOM)/HCl algorithm with simple matching distance (SMD) observation dissimilarity measure as presented in Figure 7 and Figure 7—figure supplement 1. The impact of a particular clustering variable was determined by comparing the ‘explained’ clustering variance (between-cluster to total sum-of-squares ratio) between the initial cluster structure and the structure with random resampling of the variable. Differences in the clustering variances for the most influential clustering variables (Δ clustering variance > 0) are presented in the plot. Q1, Q3: first, third quartile of anti-S1/S2 IgG titer; CKD: chronic kidney disease; GI: gastrointestinal; CVD: cardiovascular disease; PD: pulmonary disease; GITD: gastrointestinal disease.

**Figure 8—figure supplement 1.**
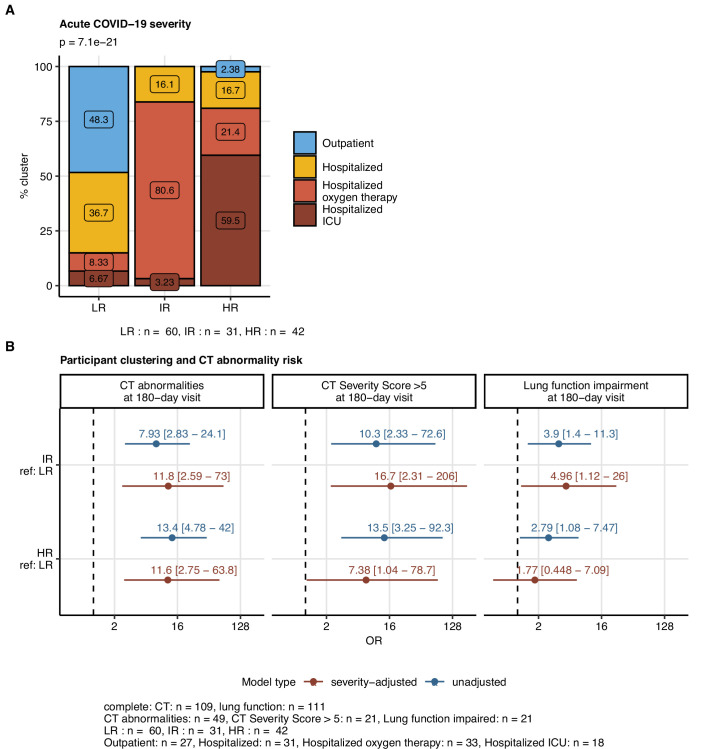
Risk of radiological lung abnormalities at the 180-day follow-up in the participant clusters. The clusters of participants were defined by non-lung function and non-computed tomography (non-CT) clinical features of acute COVID-19 and early convalescence (60-day follow-up visit, Appendix 1—table 1) with the optimally performing HCl algorithm with simple matching distance (SMD) observation dissimilarity measure as presented in Figure 7 and Figure 7—figure supplement 1. (**A**) Distribution of mild, moderate, severe, and critical acute COVID-19 cases in the participant clusters. Significance of the distribution differences was assessed with χ^2^ test. The numbers of participants assigned to the clusters are indicated under the plot. (**B**) Association of the participant cluster assignment (LR: low-risk; IR: intermediate-risk; HR: high-risk cluster) with the risk of any lung CT abnormalities and moderate-to-severe lung CT abnormalities (severity score > 5) at the 180-day follow-up visit was investigated by logistic modeling with and without inclusion of the acute COVID-19 severity effect (severity-adjusted). Odds ratio (OR) significance was determined by Wald Z test and corrected for multiple testing with the Benjamini–Hochberg method. ORs with 95% confidence intervals are presented in forest plots. Numbers of complete observations, outcome events, participants in the clusters, and the acute COVID-19 severity subsets are indicated under the plot.

### Prediction of persistent symptoms and pulmonary abnormalities by machine learning

Finally, we investigated if the 6-month follow-up outcome may be predicted by ML classifiers trained with a set of non-CT and non-LF variables recorded during acute COVID-19 and at the 60-day follow-up (Appendix 1—table 1). To this end, five technically unrelated ML classifiers were tested (Appendix 1—table 4; Kuhn, 2008)⁠: C5.0 (Quinlan, 1993)⁠, random forests (RF) (Breiman, 2001)⁠, support vector machines with radial kernel (SVM-R) (Weston and Watkins, 1998)⁠, shallow neural network (Nnet) (Ripley, 2014)⁠, and elastic net generalized linear regression (glmNet) (Friedman et al., 2010)⁠. In addition, the single classifiers with varying outcome-specific accuracy (Figure 9—figure supplement 1) were bundled into ensembles by the elastic net procedure (Figure 9—figure supplement 2, Appendix 1—table 4; Kuhn, 2008; Deane-Mayer and Knowles, 2019)⁠. Finally, the classifier and ensemble performance was investigated in the training cohort and 20-fold CV by ROC (Appendix 1—table 5).

**Figure 9.**
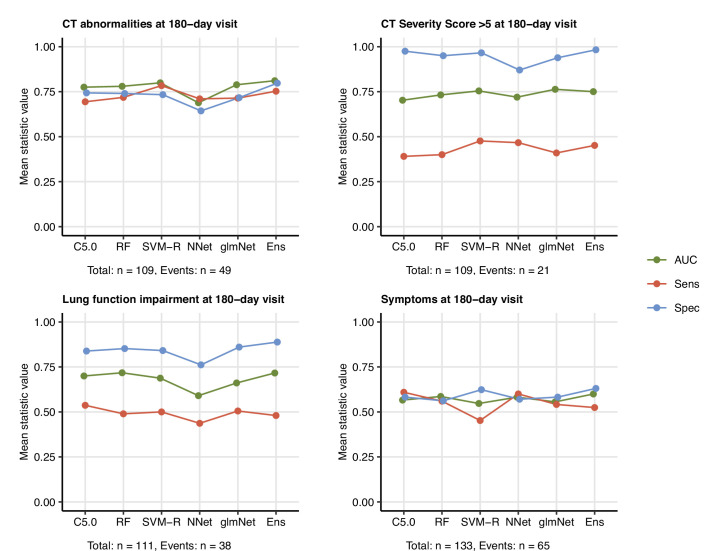
Prediction of persistent radiological lung abnormalities, functional lung impairment, and symptoms by machine learning algorithms. Single machine learning classifiers (C5.0; RF: random forests; SVM-R: support vector machines with radial kernel; NNet: neural network; glmNet: elastic net) and their ensemble (Ens) were trained in the cohort data set with 52 non-computed tomography (non-CT) and non-lung function binary explanatory variables recorded for acute COVID-19 or at the 60-day follow-up visit (Appendix 1—table 1) for predicting outcome variables at the 180-day follow-up visit (any lung CT abnormalities, moderate-to-severe lung CT abnormalities [severity score > 5], functional lung impairment, and persistent symptoms) (Appendix 1—table 4). The prediction accuracy was verified by repeated 20-fold cross-validation (five repeats). Receiver-operating characteristics (ROCs) of the algorithms in the cross-validation are presented: area under the curve (AUC), sensitivity (Sens), and specificity (Spec) (Appendix 1—table 5). The numbers of complete observations and outcome events are indicated under the plots.

**Figure 10.**
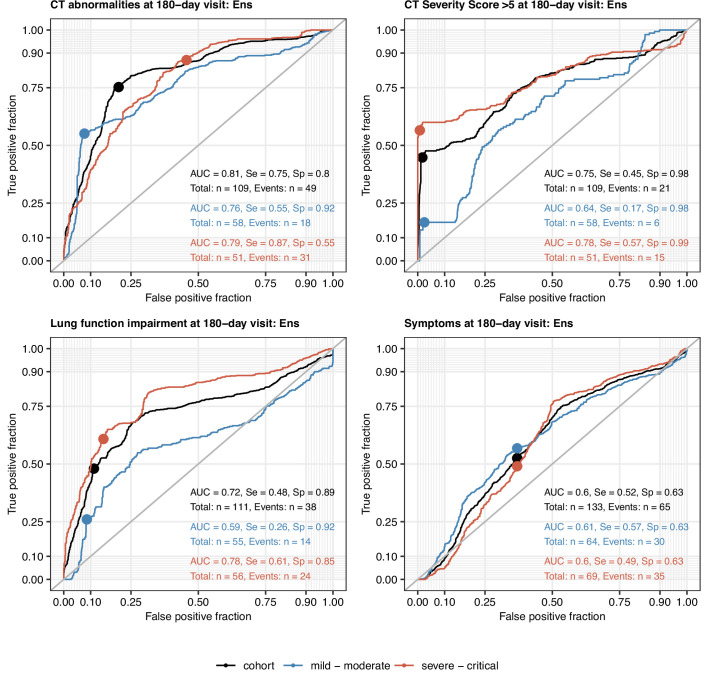
Performance of the machine learning ensemble classifier in mild-to-moderate and severe-to-critical COVID-19 convalescents. The machine learning classifier ensemble (Ens) was developed as presented in Figure 9. Its performance at predicting outcome variables at the 180-day follow-up visit (any computed tomography [CT] lung abnormalities, moderate-to-severe lung CT abnormalities [severity score > 5], functional lung impairment, and persistent symptoms) in the entire cohort, mild-to-moderate (outpatient or hospitalized without oxygen), and severe-to-critical COVID-19 convalescents (oxygen therapy or ICU) in repeated 20-fold cross-validation (five repeats) was assessed by receiver-operating characteristic (ROC) (Appendix 1—table 6). ROC curves and statistics (AUC: area under the curve; Se: sensitivity; Sp: specificity) in the cross-validation are shown. Numbers of complete observations and outcome events are indicated in the plots.

All tested ML algorithms and ensembles demonstrated good accuracy (area under the curve [AUC] > 0.78) and sensitivity (>0.84) at predicting any lung CT abnormalities at the 6-month follow-up in the study cohort serving as a training data set. Their efficiency in CV was moderate (AUC: 0.69–0.81; sensitivity: 0.69–0.78) (Figure 9, Figure 9—figure supplement 3, Appendix 1—table 5). In turn, moderate-to-severe structural lung findings were recognized with markedly lower sensitivity both in the training data set (>0.43) and the CV (0.39–0.48). Even though impaired LF and persistent symptoms were common at the 6-month follow-up in the training data set (Figures 2 and 3), nearly half of the cases were not identified by any of the tested ML algorithms and their ensembles in the CV setting (Figure 9, Figure 9—figure supplement 3, Appendix 1—table 5). The sensitivity of the ensembles and single classifiers at predicting CT and LF abnormalities was substantially better in severe and critical COVID-19 survivors than in ambulatory and moderate cases (Figure 10, Appendix 1—table 6).

**Figure 9—figure supplement 1.**
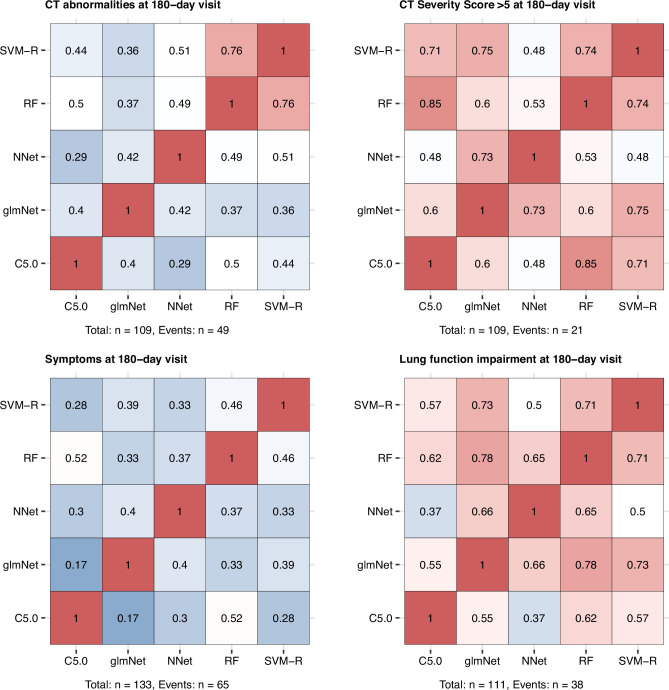
Correlation of the machine learning algorithm prediction accuracy. Machine learning classifiers (C5.0; RF: random forests; SVM-R: support vector machines with radial kernel; NNet: neural network; glmNet: elastic net) were trained in the cohort data set with 52 non-computed tomography (non-CT) and non-lung function binary explanatory variables recorded for acute COVID-19 or at the early 60-day follow-up visit (Appendix 1—table 1) for predicting outcome variables at the 180-day follow-up visit (any lung CT abnormalities, moderate-to-severe lung CT abnormalities [severity score > 5], functional lung impairment, and persistent symptoms) (Figure 9, Appendix 1—table 4). The prediction accuracy was verified by repeated 20-fold cross-validation (five repeats). Pearson’s correlation coefficients of the classifier prediction accuracy in the cross-validation are presented as heat maps. Numbers of complete observations and outcome events are indicated under the plots.

**Figure 9—figure supplement 2.**
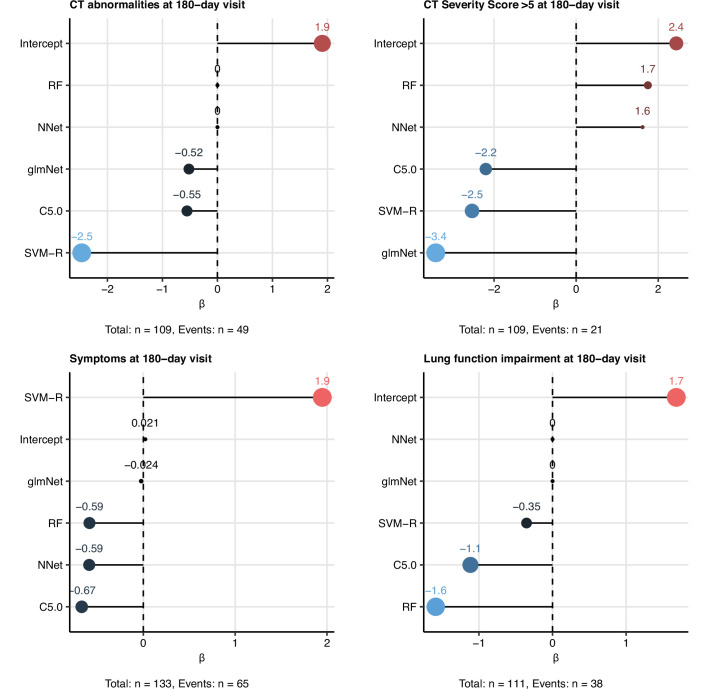
Machine learning model ensembles. Single machine learning classifiers (C5.0; RF: random forests; SVM-R: support vector machines with radial kernel; NNet: neural network; glmNet: elastic net) were trained as shown in Figure 9. The model ensembles based on the single classifiers were constructed with the glmNet procedure (Appendix 1—table 4). glmNet regression coefficients (β) are presented in the plots. Point and text color correspond to the β value. Numbers of complete observations and outcome events are indicated under the plots.

**Figure 9—figure supplement 3.**
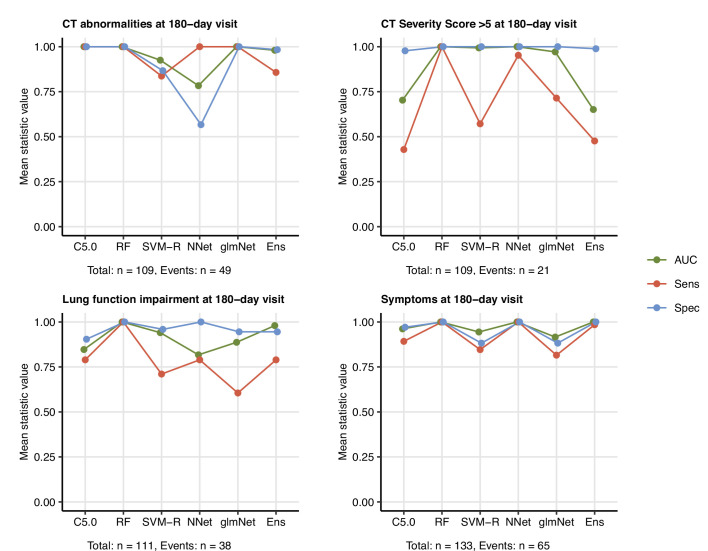
Prediction of persistent radiological lung abnormalities, functional lung impairment, and symptoms by machine learning algorithms in the training data sets. Single machine learning classifiers (C5.0; RF: random forests; SVM-R: support vector machines with radial kernel; NNet: neural network; glmNet: elastic net) and their ensembles were trained as shown in Figure 9. Performance of the classifiers in the training data sets was investigated by receiver-operating characteristic (ROC) of the algorithms (AUC: area under the curve; Sens: sensitivity; Spec: specificity, Appendix 1—table 5). Numbers of complete observations and outcome events are indicated under the plots.

**Figure 9—figure supplement 4.**
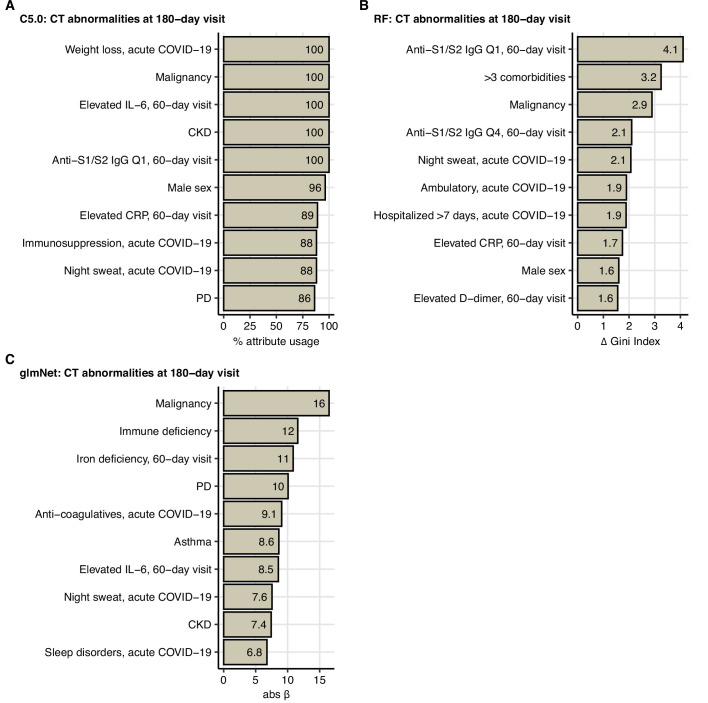
Variable importance statistics for prediction of lung computed tomography (CT) abnormalities at the 180-day follow-up by machine learning classifiers. C5.0, random forests (RF), and elastic net (glmNet) classifiers were trained as presented in Figure 9 for prediction of any lung CT abnormalities at the 180-day follow-up visit. Variable importance measures (C5.0: % attribute/variable usage in the tree model (**A**); RF: difference in Gini index (**B**); glmNet: absolute value of the regression coefficient β (**C**)) for the 10 most influential explanatory variables are presented. CKD: chronic kidney disease; Q1, Q4: first, fourth quartile of anti-S1/S2 IgG titer; PD: pulmonary disease; CKD: chronic kidney disease.

**Figure 9—figure supplement 5.**
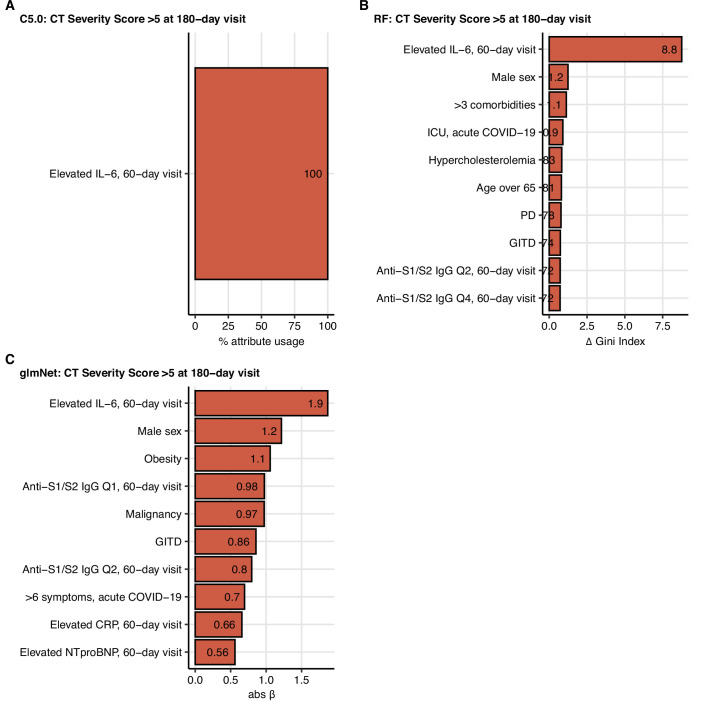
Variable importance statistics for prediction of moderate-to-severe lung computed tomography (CT) abnormalities at the 180-day follow-up by machine learning classifiers. C5.0, random forests (RF), and elastic net (glmNet) classifiers were trained as presented in Figure 9 for prediction of moderate-to-severe lung CT abnormalities (severity score > 5) at the 180-day follow-up visit. Variable importance measures (C5.0: % attribute/variable usage in the tree model (**A**); RF: difference in Gini index (**B**); glmNet: absolute value of the regression coefficient β (**C**)) for the 10 most influential explanatory variables are presented. PD: pulmonary disease; GITD: gastrointestinal disease; Q1, Q2, Q4: first, second, fourth quartile of anti-S1/S2 IgG titer.

**Figure 9—figure supplement 6.**
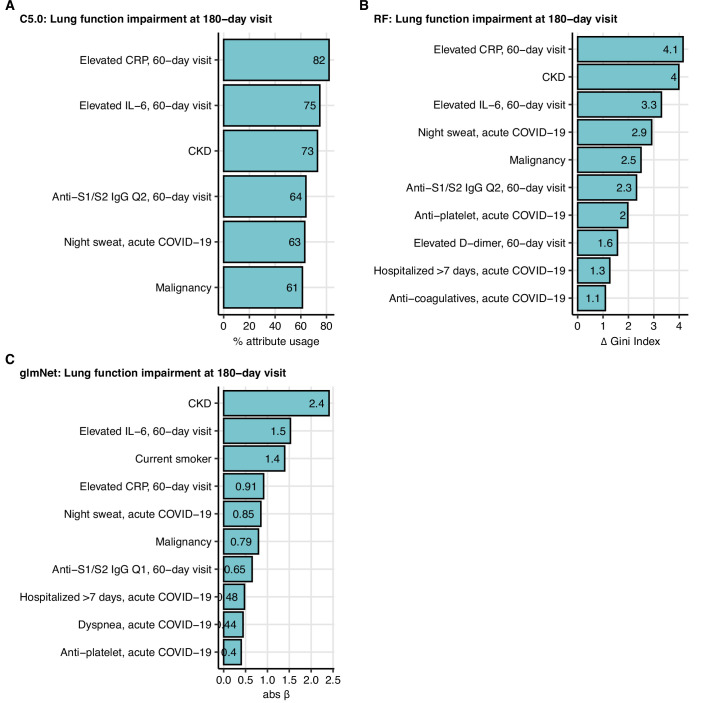
Variable importance statistics for prediction of functional lung impairment at the 180-day follow-up by machine learning classifiers. C5.0, random forests (RF), and elastic net (glmNet) classifiers were trained as presented in Figure 9 for prediction of functional lung impairment at the 180-day follow-up visit. Variable importance measures (C5.0: % attribute/variable usage in the tree model (**A**); RF: difference in Gini index (**B**); glmNet: absolute value of the regression coefficient β (**C**)) for the 10 most influential explanatory variables are presented. CKD: chronic kidney disease; Q1, Q2: first. second quartile of anti-S1/S2 IgG titer.

**Figure 9—figure supplement 7.**
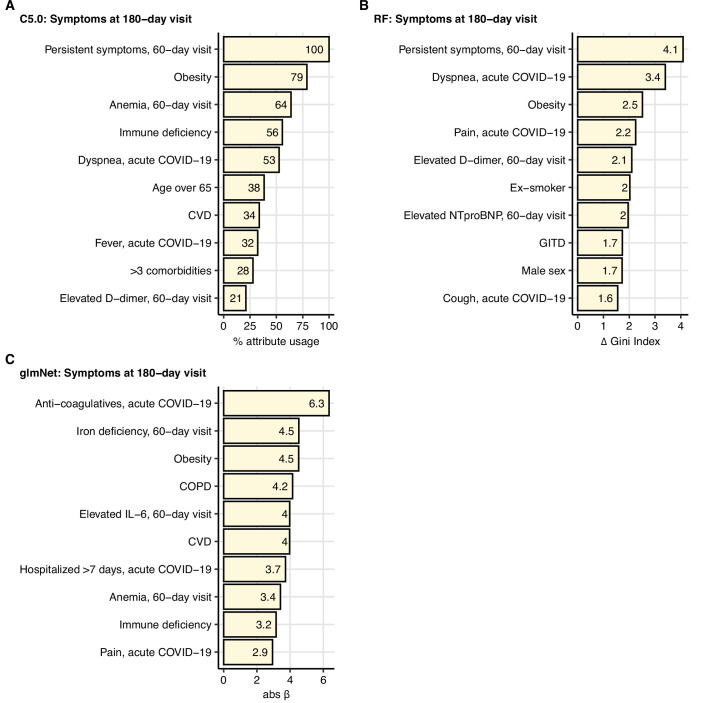
Variable importance statistics for prediction of persistent symptoms at the 180-day follow-up by machine learning classifiers. C5.0, random forests (RF), and elastic net (glmNet) classifiers were trained as presented in Figure 9 for prediction of persistent symptoms at the 180-day follow-up visit. Variable importance measures (C5.0: % attribute/variable usage in the tree model (**A**); RF: difference in Gini index (**B**); glmNet: absolute value of the regression coefficient β (**C**)) for the 10 most influential explanatory variables are presented. CVD: cardiovascular disease; GITD: gastrointestinal disease; COPD: chronic obstructive lung disease.

The most important explanatory variables for pulmonary abnormalities by three unrelated classifiers (C5.0, RF, and glmNet) included preexisting malignancy, multimorbidity, markers of systemic inflammation (IL-6 and CRP), and anti-S1/S2 antibody levels at the 60-day follow-up (Figure 9—figure supplement 4, Figure 9—figure supplement 5, Figure 9—figure supplement 6). The highly influential parameters at prediction of symptoms at the 180-day follow-up encompassed symptom presence at the 60-day follow-up, as well as obesity and dyspnea during acute COVID-19 (Figure 9—figure supplement 7).

## Discussion

Herein, we prospectively evaluated trajectories of COVID-19 recovery in an observational cohort enrolled in the Austrian CovILD study (Sonnweber et al., 2021)⁠. Despite the resolution of symptoms and pulmonary abnormalities at the 6-month follow-up in a large fraction of the study participants, the recovery pace was substantially slower in the late convalescence when compared with the first three months after diagnosis (Sonnweber et al., 2021; Huang et al., 2021a)⁠. Persistent symptoms and CT findings were detected in more than 40% and reduced LF in approximately one-third of the cohort, which is in line with recovery kinetics and signs of lung lesion chronicity reported by others (Caruso et al., 2021; Huang et al., 2021b; Huang et al., 2021a; Faverio et al., 2021; Hellemons et al., 2021; Zhou et al., 2021)⁠. By comparison, similar protracted pulmonary recovery was reported for SARS (Hui et al., 2005; Ng et al., 2004; Ngai et al., 2010; Lam et al., 2009)⁠ and non-COVID-19 acute respiratory distress syndrome (Wilcox et al., 2013; Masclans et al., 2011)⁠. Of note, treatment approaches for hospitalized patients in our cohorts and similar cohorts recruited at the pandemic onset in early 2020 (Caruso et al., 2021; Huang et al., 2021b; Huang et al., 2021a; Faverio et al., 2021; Hellemons et al., 2021)⁠ differ significantly from the current standard of care for acute COVID-19, which includes early systemic steroid use and antiviral and various immunomodulatory medications. How improved standardized therapy and anti-SARS-CoV-2 vaccination affect the clinical and pulmonary recovery needs to be investigated.

In roughly half of our study participants with abnormal lung CT findings, and especially in those with low-grade structural abnormalities, no overt LF impairment at follow-up was discerned. Still, even subclinical lung alterations may bear the potential for clinically relevant progression of interstitial lung disease (Suliman et al., 2015; Hatabu et al., 2020) requiring systematic CT and LF monitoring. Conversely, symptom persistence was weakly associated with incomplete functional or structural pulmonary recovery.

Since PASC are found in as many as 10% of COVID-19 patients (Sahanic et al., 2021; Venkatesan, 2021; Sudre et al., 2021b)⁠, robust, resource-saving tools assessing the individual risk of pulmonary complications are urgently needed (Shah et al., 2021; Raghu and Wilson, 2020)⁠. Covariates and characteristics of severe acute COVID-19 such as male sex, age, and preexisting comorbidities, hospitalization, ventilation, and ICU stay were proposed as the risk factors of persistent pulmonary impairment (Sonnweber et al., 2021; Caruso et al., 2021; Huang et al., 2021a; Faverio et al., 2021; Raghu and Wilson, 2020)⁠. However, their applicability in predicting complications of pulmonary recovery from mild or moderate COVID-19 is limited. Our results of univariate modeling, clustering, and ML prediction point towards a distinct long-term pulmonary risk phenotype that manifests during acute COVID-19 and early recovery and whose central components are protracted systemic (IL-6, CRP, anemia of inflammation) and microvascular inflammation (D-dimer), and strong humoral response (anti-S1/S2 IgG) demographic risk factors and comorbidities (Sonnweber et al., 2020)⁠. Hence, consecutive monitoring of systemic inflammatory parameters analogous to concepts of interstitial lung disease in autoimmune disorders (Khanna et al., 2020) and anti-S1/S2 antibody levels may improve identification of the individuals at risk of chronic pulmonary damage irrespective of the acute COVID-19 severity.

Clustering and ML have been employed for deep phenotyping and predicting acute and post-acute COVID-19 outcomes in multivariable data sets (Sahanic et al., 2021; Sudre et al., 2021a; Estiri et al., 2021; Demichev et al., 2021; Benito-León et al., 2021)⁠. We demonstrate that subsets of COVID-19 patients that significantly differ in the risk for long-term CT abnormalities may be defined by an easily accessible clinical parameter set available at the early post-COVID-19 assessment. This approach did not involve any CT or LF variables. Furthermore, the cluster classification correlated with the risk of long-term pulmonary abnormalities independently of the acute COVID-19 severity. Thus, these characteristics provide a useful tool for broad screening of convalescent populations, including individuals who experienced mild or moderate COVID-19.

We show that technically unrelated ML classifiers and their ensemble trained without CT and LF explanatory variables can predict lung CT findings independently of their grading at the 6-month follow-up with good specificity and sensitivity in the training collective and CV. By contrast, the more specific prediction of moderate-to-severe lung CT or risk estimation for LF deficits demonstrated a limited sensitivity. For the moderate-to-severe CT abnormalities, this can be primarily traced back to their low frequency resulting in a suboptimal classifier training, especially in CV. A substantial fraction of the participants (20.7%, n = 30) suffered from a preexisting respiratory condition (pulmonary disease, asthma, or COPD) likely paralleled by LF reduction, which possibly confounded the prediction of the post-COVID-19 LF deficits both by clustering and ML. Accumulating evidence suggests that post-acute COVID-19 symptoms are highly heterogeneous conditions with multiorgan, neurocognitive, and psychological manifestations (Sahanic et al., 2021; Evans et al., 2021; Davis et al., 2021)⁠, which may differ in risk factor constellations. This could explain why univariate modeling, clustering, and ML failed to estimate persistent symptom risk in our small study cohort. In general, the ML prediction quality may greatly benefit from a larger training data set and inclusion of additional explanatory variables such as cellular readouts of inflammation, in-depth medication, and broader acute symptom data. Nevertheless, the herein described cluster- and ML classifiers represent resource-effective tools that may assist in the screening of medical record data and identification of COVID-19 patients requiring systematic CT and LF monitoring. To facilitate the identification of patients at risk for protracted respiratory recovery and enable validation in an external collective, we implemented the clustering and prediction procedures in an open-source risk assessment application (https://im2-ibk.shinyapps.io/CovILD/).

Our study bears limitations primarily concerning the low sample size and the cross-sectional character of the trial. Because of the impaired availability of the patients and the prolonged inpatient rehabiliation, the 60- and 100-day follow-up visits in part showed a temporal overlap that may have impacted the accuracy of the longitudinal data. Missingness of the consecutive outcome variable record and the participant dropout, particularly of mild and moderate COVID-19 cases, may have also potentially confounded the participant clustering results and ML risk estimation for CT abnormalities and LF impairment since prolonged hospitalization was found to be a crucial cluster-defining and influential explanatory feature. Additionally, even though the reproducibility of the risk assessment algorithms was partially addressed by CV, cluster and ML classifiers call for verification in a larger, independent multicenter collective of COVID-19 convalescents.

In summary, in our CovILD study cohort we found a high frequency of CT and LF abnormalities and persistent symptoms at the 6-month follow-up, and a flattened recovery kinetics after 3 months post-COVID-19. Systematic risk modeling reveled a set of clinical variables linked to protracted pulmonary recovery apart from the severity of acute infection such as inflammatory markers, anti-S1/S2 IgG levels, multimorbidity, and male sex. We demonstrate that clustering and ML classifiers may help to identify individuals at risk of persistent lung lesions and to relocate medical resources to prevent long-term disability.

## Data Availability

The complete R analysis pipeline and the anonymized study data in form of stratified study variables are available as a public GitHub repository: https://github.com/PiotrTymoszuk/CovILD_6_Months (copy archived at swh:1:rev:df521ede1d284e074a0484d3e4d0ce71097d00c3). The R code for the key tools used for uni-variate modeling and model quality control (Figures 4 and 5, https://github.com/PiotrTymoszuk/lmqc; copy archived at swh:1:rev:a020119d8f23b60901115c5c2ce6f6c71998ed31), cluster analysis and its quality control (Figures 6–7, https://github.com/PiotrTymoszuk/clustering-tools-2; copy archived at swh:1:rev:64141197ca28838a8978dce9093443537157d79f) and the risk assessment applicaiton (https://github.com/PiotrTymoszuk/COVILD-recovery-assessment-app; copy archived at swh:1:rev:95f02215f4c13425d3b76f6a13b7862a53279ab9) is available at GitHub. Source data for Figures 2–10 has been included as Source data 1.

## Additional files

Transparent reporting form

Source data 1. Figure plots source data.

## Appendix 1

**Appendix 1—table 1.** Study variables. Variable: variable name in the analysis pipeline; reference time point: study visit, the variable was recorded at; label: variable label in figures and tables.

Variable	Reference time point	Label	Variable type	Stratification cutoff
sex_male_V0	Acute COVID-19	Male sex	Explanatory	
obesity_rec_V0	Acute COVID-19	Obesity	Explanatory	BMI > 30 kg/m^2^
current_smoker_V0	Acute COVID-19	Current smoker	Explanatory	
smoking_ex_V0	Acute COVID-19	Ex-smoker	Explanatory	
CVDis_rec_V0	Acute COVID-19	CVD	Explanatory	
hypertension_rec_V0	Acute COVID-19	Hypertension	Explanatory	
PDis_rec_V0	Acute COVID-19	PD	Explanatory	
COPD_rec_V0	Acute COVID-19	COPD	Explanatory	
asthma_rec_V0	Acute COVID-19	Asthma	Explanatory	
endocrine_metabolic_rec_V0	Acute COVID-19	Metabolic disorders	Explanatory	
hypercholesterolemia_rec_V0	Acute COVID-19	Hypercholesterolemia	Explanatory	
diabetes_rec_V0	Acute COVID-19	Diabetes	Explanatory	
CKDis_rec_V0	Acute COVID-19	CKD	Explanatory	
GITDis_rec_V0	Acute COVID-19	GITD	Explanatory	
malignancy_rec_V0	Acute COVID-19	Malignancy	Explanatory	
immune_deficiency_rec_V0	Acute COVID-19	Immune deficiency	Explanatory	
weight_change_rec_V0	Acute COVID-19	Weight loss, acute COVID-19	Explanatory	≥1 kg
dyspnoe_rec_V0	Acute COVID-19	Dyspnea, acute COVID-19	Explanatory	
cough_rec_V0	Acute COVID-19	Cough, acute COVID-19	Explanatory	
fever_rec_V0	Acute COVID-19	Fever, acute COVID-19	Explanatory	
night_sweat_rec_V0	Acute COVID-19	Night sweat, acute COVID-19	Explanatory	
pain_rec_V0	Acute COVID-19	Pain, acute COVID-19	Explanatory	
GI_sympt_rec_V0	Acute COVID-19	GI symptoms, acute COVID-19	Explanatory	
anosmia_rec_V0	Acute COVID-19	Anosmia, acute COVID-19	Explanatory	
ECOG_imp_rec_V0	Acute COVID-19	Impaired performance, acute COVID-19	Explanatory	ECOG ≥ 1
sleep_disorder_rec_V0	Acute COVID-19	Sleep disorders, acute COVID-19	Explanatory	
treat_antiinfec_rec_V0	Acute COVID-19	Anti-infectives, acute COVID-19	Explanatory	
treat_antiplat_rec_V0	Acute COVID-19	Antiplatelet, acute COVID-19	Explanatory	
treat_anticoag_rec_V0	Acute COVID-19	Anticoagulatives, acute COVID-19	Explanatory	
treat_immunosuppr_rec_V0	Acute COVID-19	Immunosuppression, acute COVID-19	Explanatory	
anemia_rec_V1	60-day follow-up	Anemia, 60-day visit	Explanatory	Male: Hb < 14 g/dL; female: Hb <12 g/dL
ferr_elv_rec_V1	60-day follow-up	Elevated ferritin, 60-day visit	Explanatory	Male: > 300 ng/mL; female: > 150 ng/mL
NTelv_rec_V1	60-day follow-up	Elevated NTproBNP, 60-day visit	Explanatory	>125 pg/mL
Ddimerelv_rec_V1	60-day follow-up	Elevated D-dimer, 60-day visit	Explanatory	>500 pg/mL FEU
CRP_elv_rec_V1	60-day follow-up	Elevated CRP, 60-day visit	Explanatory	>0.5 mg/dL
IL6_elv_rec_V1	60-day follow-up	Elevated IL-6, 60-day visit	Explanatory	>7 pg/mL
iron_deficiency_30_rec_V1	60-day follow-up	Iron deficiency, 60-day visit	Explanatory	TF-saturation < 15%
age_65_V0	Acute COVID-19	Age over 65	Explanatory	>65 years
hosp_7d_V0	Acute COVID-19	Hospitalized > 7 days, acute COVID-19	Explanatory	>7 days
comorb_present_V0	Acute COVID-19	Any comorbidity	Explanatory	>0 comorbidities
comorb_3_V0	Acute COVID-19	>3 comorbidities	Explanatory	>3 comorbidities
overweight_V0	Acute COVID-19	Overweight or obesity	Explanatory	BMI > 25 kg/m^2^
sympt_6_V0	Acute COVID-19	>6 symptoms, acute COVID-19	Explanatory	>6 symptoms
sympt_present_V1	60-day follow-up	Persistent symptoms, 60-day visit	Explanatory	>0 symptoms at 180-day visit
ab_0_V1	60-day follow-up	Anti-S1/S2 IgG Q1, 60-day visit	Explanatory	(0, 312] BAU/mL
ab_25_V1	60-day follow-up	Anti-S1/S2 IgG Q2, 60-day visit	Explanatory	(312, 644] BAU/mL
ab_50_V1	60-day follow-up	Anti-S1/S2 IgG Q3, 60-day visit	Explanatory	(644, 975] BAU/mL
ab_75_V1	60-day follow-up	Anti-S1/S2 IgG Q4, 60-day visit	Explanatory	> 975 BAU/mL
pat_group_G1_V0	Acute COVID-19	Ambulatory, acute COVID-19	Explanatory	
pat_group_G2_V0	Acute COVID-19	Hospitalized, acute COVID-19	Explanatory	
pat_group_G3_V0	Acute COVID-19	Oxygen therapy, acute COVID-19	Explanatory	
pat_group_G4_V0	Acute COVID-19	ICU, acute COVID-19	Explanatory	
CT_findings_V3	180-day follow-up	CT abnormalities at 180-day visit	Outcome	
CT_sev_low_V3	180-day follow-up	CT severity score 1–5 at 180-day visit	Outcome	
CTsevabove5_V3	180-day follow-up	CT severity score >5 at 180-day visit	Outcome	
sympt_present_V3	180-day follow-up	Symptoms at 180-day visit	Outcome	
lung_function_impaired_V3	180-day follow-up	Lung function impairment at 180-day visit	Outcome	

CVD = cardiovascular disease; PD = pulmonary disease; COPD = chronic obstructive pulmonary disease; CKD = chronic kidney disease; GITD = gastrointestinal disease; GI = gastrointestinal; CRP = C-reactive protein; ICU = intensive care unit; CT = computed tomography; BMI = body mass index; BAU = binding antibody unit.

**Appendix 1—table 2.** Results of univariate risk modeling. Outcome: outcome variable at the 180-day follow-up visit; covariate: explanatory variable; baseline: reference level of the explanatory variable; OR: odds ratios with 95% confidence intervals; pFDR: significanct p-value corrected for multiple testing with the Benjamini–Hochberg method (FDR: false discovery rate).

Outcome	Covariate	Baseline	Complete cases	OR	pFDR
CT abnormalities at 180-day visit	Male sex, n = 63	No male sex, n = 55	118	3.79 [1.77–8.44]	p=0.01
CT abnormalities at 180-day visit	Obesity, n = 22	No obesity, n = 96	118	1.07 [0.415–2.72]	ns (p=0.9)
CT abnormalities at 180-day visit	Current smoker, n = 4	No current smoker, n = 114	118	0.412 [0.02–3.33]	ns (p=0.51)
CT abnormalities at 180-day visit	Ex-smoker, n = 48	No ex-smoker, n = 70	118	1.5 [0.716–3.16]	ns (p=0.36)
CT abnormalities at 180-day visit	CVD, n = 45	No CVD, n = 73	118	3.36 [1.57–7.43]	p=0.012
CT abnormalities at 180-day visit	Hypertension, n = 34	No hypertension, n = 84	118	3.97 [1.73–9.54]	p=0.01
CT abnormalities at 180-day visit	PD, n = 24	No PD, n = 94	118	2.06 [0.837–5.25]	ns (p=0.2)
CT abnormalities at 180-day visit	COPD, n = 6	No COPD, n = 112	118	2.67 [0.499–19.8]	ns (p=0.34)
CT abnormalities at 180-day visit	Asthma, n = 9	No asthma, n = 109	118	1.02 [0.24–4.04]	ns (p=0.99)
CT abnormalities at 180-day visit	Metabolic disorders, n = 50	No metabolic disorders, n = 68	118	3.14 [1.48–6.81]	p=0.017
CT abnormalities at 180-day visit	Hypercholesterolemia, n = 22	No hypercholesterolemia, n = 96	118	2.67 [1.04–7.27]	ns (p=0.093)
CT abnormalities at 180-day visit	Diabetes, n = 18	No diabetes, n = 100	118	4.07 [1.41–13.5]	p=0.041
CT abnormalities at 180-day visit	GITD, n = 17	No GITD, n = 101	118	3.66 [1.25–12.2]	ns (p=0.061)
CT abnormalities at 180-day visit	Malignancy, n = 13	No malignancy, n = 105	118	19.5 [3.63–362]	p=0.021
CT abnormalities at 180-day visit	Immune deficiency, n = 5	No immune deficiency, n = 113	118	1.96 [0.313–15.3]	ns (p=0.53)
CT abnormalities at 180-day visit	Weight loss, acute COVID-19, n = 84	No weight loss, acute COVID-19, n = 34	118	4.45 [1.83–12.1]	p=0.011
CT abnormalities at 180-day visit	Dyspnea, acute COVID-19, n = 81	No dyspnea, acute COVID-19, n = 37	118	1.45 [0.661–3.27]	ns (p=0.43)
CT abnormalities at 180-day visit	Cough, acute COVID-19, n = 83	No cough, acute COVID-19, n = 35	118	1.07 [0.484–2.41]	ns (p=0.89)
CT abnormalities at 180-day visit	Fever, acute COVID-19, n = 83	No fever, acute COVID-19, n = 35	118	2.56 [1.12–6.21]	ns (p=0.072)
CT abnormalities at 180-day visit	Night sweat, acute COVID-19, n = 74	No night sweat, acute COVID-19, n = 44	118	1.93 [0.902–4.26]	ns (p=0.17)
CT abnormalities at 180-day visit	Pain, acute COVID-19, n = 65	No pain, acute COVID-19, n = 53	118	0.339 [0.157–0.713]	p=0.021
CT abnormalities at 180-day visit	GI symptoms, acute COVID-19, n = 47	No GI symptoms, acute COVID-19, n = 71	118	0.675 [0.316–1.42]	ns (p=0.38)
CT abnormalities at 180-day visit	Anosmia, acute COVID-19, n = 53	No anosmia, acute COVID-19, n = 65	118	1.09 [0.526–2.28]	ns (p=0.85)
CT abnormalities at 180-day visit	Impaired performance, acute COVID-19, n = 106	No impaired performance, acute COVID-19, n = 12	118	1.12 [0.335–3.98]	ns (p=0.89)
CT abnormalities at 180-day visit	Sleep disorders, acute COVID-19, n = 40	No sleep disorders, acute COVID-19, n = 77	117	0.887 [0.407–1.91]	ns (p=0.82)
CT abnormalities at 180-day visit	Anti-infectives, acute COVID-19, n = 64	No anti-infectives, acute COVID-19, n = 54	118	3.56 [1.67–7.9]	p=0.01
CT abnormalities at 180-day visit	Antiplatelet, acute COVID-19, n = 12	No antiplatelet, acute COVID-19, n = 106	118	4.4 [1.23–20.7]	ns (p=0.077)
CT abnormalities at 180-day visit	Anticoagulatives, acute COVID-19, n = 4	No anticoagulatives, acute COVID-19, n = 114	118	3.98 [0.493–81.8]	ns (p=0.32)
CT abnormalities at 180-day visit	Immunosuppression, acute COVID-19, n = 20	No immunosuppression, acute COVID-19, n = 98	118	6.89 [2.32–25.5]	p=0.01
CT abnormalities at 180-day visit	Anemia, 60-day visit, n = 10	No anemia, 60-day visit, n = 108	118	5.82 [1.38–39.8]	ns (p=0.072)
CT abnormalities at 180-day visit	Elevated ferritin, 60-day visit, n = 20	No elevated ferritin, 60-day visit, n = 98	118	2.18 [0.825–6.01]	ns (p=0.2)
CT abnormalities at 180-day visit	Elevated NTproBNP, 60-day visit, n = 38	No elevated NTproBNP, 60-day visit, n = 80	118	2.29 [1.05–5.1]	ns (p=0.084)
CT abnormalities at 180-day visit	Elevated D-dimer, 60-day visit, n = 49	No elevated D-dimer, 60-day visit, n = 69	118	2.9 [1.37–6.28]	p=0.023
CT abnormalities at 180-day visit	Elevated CRP, 60-day visit, n = 18	No elevated CRP, 60-day visit, n = 100	118	5.71 [1.89–21.3]	p=0.019
CT abnormalities at 180-day visit	Elevated IL-6, 60-day visit, n = 11	No elevated IL-6, 60-day visit, n = 107	118	15.5 [2.81–289]	p=0.036
CT abnormalities at 180-day visit	Iron deficiency, 60-day visit, n = 6	No iron deficiency, 60-day visit, n = 112	118	0.239 [0.0123–1.55]	ns (p=0.29)
CT abnormalities at 180-day visit	Age over 65, n = 32	No age over 65, n = 86	118	2.81 [1.23–6.66]	p=0.045
CT abnormalities at 180-day visit	Hospitalized >7 days, acute COVID-19, n = 59	No hospitalized >7 days, acute COVID-19, n = 59	118	4.93 [2.28–11.1]	p=0.0026
CT abnormalities at 180-day visit	Any comorbidity, n = 90	No any comorbidity, n = 28	118	6.86 [2.41–24.8]	p=0.01
CT abnormalities at 180-day visit	>3 comorbidities, n = 37	No >3 comorbidities, n = 81	118	6.05 [2.62–14.9]	p=0.0026
CT abnormalities at 180-day visit	Overweight or obesity, n = 72	No overweight or obesity, n = 46	118	1.61 [0.762–3.48]	ns (p=0.3)
CT abnormalities at 180-day visit	>6 symptoms, acute COVID-19, n = 33	No >6 symptoms, acute COVID-19, n = 85	118	0.767 [0.333–1.73]	ns (p=0.59)
CT abnormalities at 180-day visit	Persistent symptoms, 60-day visit, n = 93	No persistent symptoms, 60-day visit, n = 25	118	1.91 [0.769–5.08]	ns (p=0.26)
CT abnormalities at 180-day visit	Anti-S1/S2 IgG Q1, 60-day visit, n = 31	No anti-S1/S2 IgG Q1, 60-day visit, n = 79	110	0.0769 [0.0173–0.24]	p=0.0026
CT abnormalities at 180-day visit	Anti-S1/S2 IgG Q2, 60-day visit, n = 30	No anti-S1/S2 IgG Q2, 60-day visit, n = 80	110	1.12 [0.481–2.62]	ns (p=0.83)
CT abnormalities at 180-day visit	Anti-S1/S2 IgG Q3, 60-day visit, n = 27	No anti-S1/S2 IgG Q3, 60-day visit, n = 83	110	1.8 [0.753–4.4]	ns (p=0.28)
CT abnormalities at 180-day visit	Anti-S1/S2 IgG Q4, 60-day visit, n = 22	No anti-S1/S2 IgG Q4, 60-day visit, n = 88	110	5.95 [2.13–19.5]	p=0.01
CT abnormalities at 180-day visit	Ambulatory, acute COVID-19, n = 33	No ambulatory, acute COVID-19, n = 85	118	0.106 [0.0296–0.299]	p=0.0026
CT abnormalities at 180-day visit	Hospitalized, acute COVID-19, n = 33	No hospitalized, acute COVID-19, n = 85	118	1.28 [0.569–2.88]	ns (p=0.61)
CT abnormalities at 180-day visit	Oxygen therapy, acute COVID-19, n = 33	No oxygen therapy, acute COVID-19, n = 85	118	1.52 [0.676–3.43]	ns (p=0.38)
CT abnormalities at 180-day visit	ICU, acute COVID-19, n = 19	No ICU, acute COVID-19, n = 99	118	6.28 [2.1–23.3]	p=0.012
CT severity score >5 at 180-day visit	Male sex, n = 63	No male sex, n = 55	118	5.1 [1.75–18.7]	p=0.01
CT severity score >5 at 180-day visit	Obesity, n = 22	No obesity, n = 96	118	0.38 [0.0577–1.46]	ns (p=0.26)
CT severity score >5 at 180-day visit	Current smoker, n = 4	No current smoker, n = 114	118	1.48 [0.0711–12.2]	ns (p=0.77)
CT severity score >5 at 180-day visit	Ex-smoker, n = 48	No ex-smoker, n = 70	118	1.59 [0.623–4.09]	ns (p=0.37)
CT severity score >5 at 180-day visit	CVD, n = 45	No CVD, n = 73	118	4.71 [1.8–13.5]	p=0.0042
CT severity score >5 at 180-day visit	Hypertension, n = 34	No hypertension, n = 84	118	3.17 [1.21–8.38]	p=0.029
CT severity score >5 at 180-day visit	PD, n = 24	No PD, n = 94	118	2.17 [0.735–6.02]	ns (p=0.18)
CT severity score >5 at 180-day visit	COPD, n = 6	No COPD, n = 112	118	2.3 [0.304–12.7]	ns (p=0.39)
CT severity score >5 at 180-day visit	Asthma, n = 9	No asthma, n = 109	118	2.37 [0.468–9.85]	ns (p=0.29)
CT severity score >5 at 180-day visit	Metabolic disorders, n = 50	No metabolic disorders, n = 68	118	2.92 [1.14–7.95]	p=0.045
CT severity score >5 at 180-day visit	Hypercholesterolemia, n = 22	No hypercholesterolemia, n = 96	118	2.52 [0.845–7.12]	ns (p=0.12)
CT severity score >5 at 180-day visit	Diabetes, n = 18	No diabetes, n = 100	118	2.63 [0.816–7.87]	ns (p=0.12)
CT severity score >5 at 180-day visit	CKD, n = 6	No CKD, n = 112	118	4.89 [0.851–28.3]	ns (p=0.091)
CT severity score >5 at 180-day visit	GITD, n = 17	No GITD, n = 101	118	2.9 [0.892–8.83]	ns (p=0.092)
CT severity score >5 at 180-day visit	Malignancy, n = 13	No malignancy, n = 105	118	0.333 [0.0178–1.84]	ns (p=0.35)
CT severity score >5 at 180-day visit	Immune deficiency, n = 5	No immune deficiency, n = 113	118	7.42 [1.16–59.3]	ns (p=0.052)
CT severity score >5 at 180-day visit	Weight loss, acute COVID-19, n = 84	No weight loss, acute COVID-19, n = 34	118	3.02 [0.939–13.5]	ns (p=0.13)
CT severity score >5 at 180-day visit	Dyspnea, acute COVID-19, n = 81	No dyspnea, acute COVID-19, n = 37	118	1.7 [0.609–5.54]	ns (p=0.38)
CT severity score >5 at 180-day visit	Cough, acute COVID-19, n = 83	No cough, acute COVID-19, n = 35	118	0.537 [0.206–1.44]	ns (p=0.25)
CT severity score >5 at 180-day visit	Fever, acute COVID-19, n = 83	No fever, acute COVID-19, n = 35	118	2.15 [0.727–7.9]	ns (p=0.24)
CT severity score >5 at 180-day visit	Night sweat, acute COVID-19, n = 74	No night sweat, acute COVID-19, n = 44	118	2.33 [0.84–7.55]	ns (p=0.17)
CT severity score >5 at 180-day visit	Pain, acute COVID-19, n = 65	No pain, acute COVID-19, n = 53	118	0.495 [0.187–1.26]	ns (p=0.18)
CT severity score >5 at 180-day visit	GI symptoms, acute COVID-19, n = 47	No GI symptoms, acute COVID-19, n = 71	118	0.503 [0.168–1.34]	ns (p=0.23)
CT severity score >5 at 180-day visit	Anosmia, acute COVID-19, n = 53	No anosmia, acute COVID-19, n = 65	118	1.61 [0.634–4.16]	ns (p=0.36)
CT severity score >5 at 180-day visit	Impaired performance, acute COVID-19, n = 106	No impaired performance, acute COVID-19, n = 12	118	2.72 [0.486–51]	ns (p=0.39)
CT severity score >5 at 180-day visit	Sleep disorders, acute COVID-19, n = 40	No sleep disorders, acute COVID-19, n = 77	117	1.13 [0.412–2.91]	ns (p=0.84)
CT severity score >5 at 180-day visit	Anti-infectives, acute COVID-19, n = 64	No anti-infectives, acute COVID-19, n = 54	118	4.89 [1.68–17.9]	p=0.012
CT severity score >5 at 180-day visit	Antiplatelet, acute COVID-19, n = 12	No antiplatelet, acute COVID-19, n = 106	118	3.74 [1.01–13.2]	ns (p=0.06)
CT severity score >5 at 180-day visit	Anticoagulatives, acute COVID-19, n = 4	No anticoagulatives, acute COVID-19, n = 114	118	4.7 [0.538–41.1]	ns (p=0.17)
CT severity score >5 at 180-day visit	Immunosuppression, acute COVID-19, n = 20	No immunosuppression, acute COVID-19, n = 98	118	5.35 [1.85–15.6]	p=0.0036
CT severity score >5 at 180-day visit	Anemia, 60-day visit, n = 10	No anemia, 60-day visit, n = 108	118	8.62 [2.23–37.1]	p=0.0039
CT severity score >5 at 180-day visit	Elevated ferritin, 60-day visit, n = 20	No elevated ferritin, 60-day visit, n = 98	118	2.2 [0.693–6.42]	ns (p=0.2)
CT severity score >5 at 180-day visit	Elevated NTproBNP, 60-day visit, n = 38	No elevated NTproBNP, 60-day visit, n = 80	118	3.23 [1.25–8.55]	p=0.026
CT severity score >5 at 180-day visit	Elevated D-dimer, 60-day visit, n = 49	No elevated D-dimer, 60-day visit, n = 69	118	2.41 [0.945–6.38]	ns (p=0.096)
CT severity score >5 at 180-day visit	Elevated CRP, 60-day visit, n = 18	No elevated CRP, 60-day visit, n = 100	118	4.91 [1.63–14.7]	p=0.0075
CT severity score >5 at 180-day visit	Elevated IL-6, 60-day visit, n = 11	No elevated IL-6, 60-day visit, n = 107	118	32.5 [7.43–230]	p=7.5e-05
CT severity score >5 at 180-day visit	Iron deficiency, 60-day visit, n = 6	No iron deficiency, 60-day visit, n = 112	118	0.867 [0.044–5.75]	ns (p=0.92)
CT severity score >5 at 180-day visit	Age over 65, n = 32	No age over 65, n = 86	118	2.8 [1.05–7.4]	ns (p=0.055)
CT severity score >5 at 180-day visit	Hospitalized >7 days, acute COVID-19, n = 59	No hospitalized >7 days, acute COVID-19, n = 59	118	4.37 [1.58–14.2]	p=0.012
CT severity score >5 at 180-day visit	Any comorbidity, n = 90	No any comorbidity, n = 28	118	8.22 [1.59–151]	ns (p=0.065)
CT severity score >5 at 180-day visit	>3 comorbidities, n = 37	No >3 comorbidities, n = 81	118	5.55 [2.11–15.5]	p=0.0013
CT severity score >5 at 180-day visit	Overweight or obesity, n = 72	No overweight or obesity, n = 46	118	0.72 [0.282–1.87]	ns (p=0.53)
CT severity score >5 at 180-day visit	>6 symptoms, acute COVID-19, n = 33	No >6 symptoms, acute COVID-19, n = 85	118	1.26 [0.438–3.35]	ns (p=0.69)
CT severity score >5 at 180-day visit	Persistent symptoms, 60-day visit, n = 93	No persistent symptoms, 60-day visit, n = 25	118	3.15 [0.831–20.7]	ns (p=0.18)
CT severity score >5 at 180-day visit	Anti-S1/S2 IgG Q2, 60-day visit, n = 30	No anti-S1/S2 IgG Q2, 60-day visit, n = 80	110	1.87 [0.666–5.07]	ns (p=0.26)
CT severity score >5 at 180-day visit	Anti-S1/S2 IgG Q3, 60-day visit, n = 27	No anti-S1/S2 IgG Q3, 60-day visit, n = 83	110	0.675 [0.18–2.05]	ns (p=0.55)
CT severity score >5 at 180-day visit	Anti-S1/S2 IgG Q4, 60-day visit, n = 22	No anti-S1/S2 IgG Q4, 60-day visit, n = 88	110	4.38 [1.53–12.6]	p=0.01
CT severity score >5 at 180-day visit	Ambulatory, acute COVID-19, n = 33	No ambulatory, acute COVID-19, n = 85	118	0.0952 [0.0052–0.488]	p=0.039
CT severity score >5 at 180-day visit	Hospitalized, acute COVID-19, n = 33	No hospitalized, acute COVID-19, n = 85	118	0.714 [0.218–2.01]	ns (p=0.58)
CT severity score >5 at 180-day visit	Oxygen therapy, acute COVID-19, n = 33	No oxygen therapy, acute COVID-19, n = 85	118	0.958 [0.316–2.61]	ns (p=0.95)
CT severity score >5 at 180-day visit	ICU, acute COVID-19, n = 19	No ICU, acute COVID-19, n = 99	118	8.06 [2.75–24.5]	p=0.00035
Symptoms at 180-day visit	Male sex, n = 82	No male sex, n = 63	145	0.701 [0.361–1.35]	ns (p=0.97)
Symptoms at 180-day visit	Obesity, n = 28	No obesity, n = 117	145	0.42 [0.169–0.982]	ns (p=0.84)
Symptoms at 180-day visit	Current smoker, n = 4	No current smoker, n = 141	145	3.22 [0.401–66]	ns (p=0.97)
Symptoms at 180-day visit	Ex-smoker, n = 57	No ex-smoker, n = 88	145	1.27 [0.654–2.49]	ns (p=0.97)
Symptoms at 180-day visit	CVD, n = 58	No CVD, n = 87	145	0.851 [0.436–1.66]	ns (p=0.97)
Symptoms at 180-day visit	Hypertension, n = 44	No hypertension, n = 101	145	0.931 [0.456–1.89]	ns (p=0.97)
Symptoms at 180-day visit	PD, n = 27	No PD, n = 118	145	1.38 [0.598–3.26]	ns (p=0.97)
Symptoms at 180-day visit	COPD, n = 8	No COPD, n = 137	145	1.04 [0.238–4.58]	ns (p=0.97)
Symptoms at 180-day visit	Asthma, n = 10	No asthma, n = 135	145	1.05 [0.279–3.92]	ns (p=0.97)
Symptoms at 180-day visit	Metabolic disorders, n = 63	No metabolic disorders, n = 82	145	1.02 [0.527–1.96]	ns (p=0.97)
Symptoms at 180-day visit	Hypercholesterolemia, n = 27	No hypercholesterolemia, n = 118	145	0.55 [0.226–1.28]	ns (p=0.97)
Symptoms at 180-day visit	Diabetes, n = 24	No diabetes, n = 121	145	1.05 [0.434–2.54]	ns (p=0.97)
Symptoms at 180-day visit	CKD, n = 10	No CKD, n = 135	145	1.62 [0.442–6.56]	ns (p=0.97)
Symptoms at 180-day visit	GITD, n = 20	No GITD, n = 125	145	1.68 [0.649–4.55]	ns (p=0.97)
Symptoms at 180-day visit	Malignancy, n = 17	No malignancy, n = 128	145	0.7 [0.241–1.94]	ns (p=0.97)
Symptoms at 180-day visit	Immune deficiency, n = 9	No immune deficiency, n = 136	145	0.824 [0.197–3.24]	ns (p=0.97)
Symptoms at 180-day visit	Weight loss, acute COVID-19, n = 106	No weight loss, acute COVID-19, n = 39	145	1.34 [0.644–2.84]	ns (p=0.97)
Symptoms at 180-day visit	Dyspnea, acute COVID-19, n = 98	No dyspnea, acute COVID-19, n = 47	145	2.84 [1.39–6.04]	ns (p=0.2)
Symptoms at 180-day visit	Cough, acute COVID-19, n = 102	No cough, acute COVID-19, n = 43	145	1.97 [0.96–4.17]	ns (p=0.88)
Symptoms at 180-day visit	Fever, acute COVID-19, n = 106	No fever, acute COVID-19, n = 39	145	1.17 [0.559–2.45]	ns (p=0.97)
Symptoms at 180-day visit	Night sweat, acute COVID-19, n = 92	No night sweat, acute COVID-19, n = 53	145	1.42 [0.723–2.83]	ns (p=0.97)
Symptoms at 180-day visit	Pain, acute COVID-19, n = 78	No pain, acute COVID-19, n = 67	145	1.92 [0.993–3.75]	ns (p=0.84)
Symptoms at 180-day visit	GI symptoms, acute COVID-19, n = 59	No GI symptoms, acute COVID-19, n = 86	145	1.27 [0.656–2.48]	ns (p=0.97)
Symptoms at 180-day visit	Anosmia, acute COVID-19, n = 62	No anosmia, acute COVID-19, n = 83	145	1.69 [0.874–3.31]	ns (p=0.96)
Symptoms at 180-day visit	Impaired performance, acute COVID-19, n = 132	No impaired performance, acute COVID-19, n = 13	145	1.13 [0.358–3.69]	ns (p=0.97)
Symptoms at 180-day visit	Sleep disorders, acute COVID-19, n = 56	No sleep disorders, acute COVID-19, n = 88	144	1.38 [0.708–2.73]	ns (p=0.97)
Symptoms at 180-day visit	Anti-infectives, acute COVID-19, n = 78	No anti-infectives, acute COVID-19, n = 67	145	0.701 [0.362–1.35]	ns (p=0.97)
Symptoms at 180-day visit	Antiplatelet, acute COVID-19, n = 22	No antiplatelet, acute COVID-19, n = 123	145	1.05 [0.42–2.63]	ns (p=0.97)
Symptoms at 180-day visit	Anticoagulatives, acute COVID-19, n = 9	No anticoagulatives, acute COVID-19, n = 136	145	2.18 [0.553–10.7]	ns (p=0.97)
Symptoms at 180-day visit	Immunosuppression, acute COVID-19, n = 27	No immunosuppression, acute COVID-19, n = 118	145	1.38 [0.598–3.26]	ns (p=0.97)
Symptoms at 180-day visit	Anemia, 60-day visit, n = 16	No anemia, 60-day visit, n = 129	145	0.591 [0.191–1.69]	ns (p=0.97)
Symptoms at 180-day visit	Elevated ferritin, 60-day visit, n = 26	No elevated ferritin, 60-day visit, n = 118	144	1.29 [0.551–3.07]	ns (p=0.97)
Symptoms at 180-day visit	Elevated NTproBNP, 60-day visit, n = 52	No elevated NTproBNP, 60-day visit, n = 93	145	1.96 [0.987–3.94]	ns (p=0.84)
Symptoms at 180-day visit	Elevated D-dimer, 60-day visit, n = 60	No elevated D-dimer, 60-day visit, n = 85	145	1.7 [0.874–3.33]	ns (p=0.96)
Symptoms at 180-day visit	Elevated CRP, 60-day visit, n = 23	No elevated CRP, 60-day visit, n = 122	145	1.16 [0.475–2.88]	ns (p=0.97)
Symptoms at 180-day visit	Elevated IL-6, 60-day visit, n = 17	No elevated IL-6, 60-day visit, n = 128	145	0.529 [0.173–1.48]	ns (p=0.97)
Symptoms at 180-day visit	Iron deficiency, 60-day visit, n = 6	No iron deficiency, 60-day visit, n = 138	144	2.18 [0.412–16.1]	ns (p=0.97)
Symptoms at 180-day visit	Age over 65, n = 43	No age over 65, n = 102	145	1.69 [0.827–3.51]	ns (p=0.97)
Symptoms at 180-day visit	Hospitalized >7 days, acute COVID-19, n = 80	No hospitalized >7 days, acute COVID-19, n = 65	145	1.1 [0.569–2.12]	ns (p=0.97)
Symptoms at 180-day visit	Any comorbidity, n = 112	No any comorbidity, n = 33	145	1.03 [0.47–2.24]	ns (p=0.97)
Symptoms at 180-day visit	>3 comorbidities, n = 47	No >3 comorbidities, n = 98	145	1.46 [0.727–2.95]	ns (p=0.97)
Symptoms at 180-day visit	Overweight or obesity, n = 86	No overweight or obesity, n = 59	145	0.7 [0.358–1.36]	ns (p=0.97)
Symptoms at 180-day visit	>6 symptoms, acute COVID-19, n = 42	No >6 symptoms, acute COVID-19, n = 103	145	1.82 [0.885–3.82]	ns (p=0.96)
Symptoms at 180-day visit	Persistent symptoms, 60-day visit, n = 115	No persistent symptoms, 60-day visit, n = 30	145	4.12 [1.71–11.1]	ns (p=0.2)
Symptoms at 180-day visit	Anti-S1/S2 IgG Q1, 60-day visit, n = 34	No anti-S1/S2 IgG Q1, 60-day visit, n = 100	134	1.04 [0.476–2.28]	ns (p=0.97)
Symptoms at 180-day visit	Anti-S1/S2 IgG Q2, 60-day visit, n = 33	No anti-S1/S2 IgG Q2, 60-day visit, n = 101	134	1.13 [0.512–2.49]	ns (p=0.97)
Symptoms at 180-day visit	Anti-S1/S2 IgG Q3, 60-day visit, n = 34	No anti-S1/S2 IgG Q3, 60-day visit, n = 100	134	0.646 [0.289–1.41]	ns (p=0.97)
Symptoms at 180-day visit	Anti-S1/S2 IgG Q4, 60-day visit, n = 33	No anti-S1/S2 IgG Q4, 60-day visit, n = 101	134	1.32 [0.603–2.95]	ns (p=0.97)
Symptoms at 180-day visit	Ambulatory, acute COVID-19, n = 36	No ambulatory, acute COVID-19, n = 109	145	0.911 [0.426–1.94]	ns (p=0.97)
Symptoms at 180-day visit	Hospitalized, acute COVID-19, n = 37	No hospitalized, acute COVID-19, n = 108	145	0.983 [0.463–2.08]	ns (p=0.97)
Symptoms at 180-day visit	Oxygen therapy, acute COVID-19, n = 40	No oxygen therapy, acute COVID-19, n = 105	145	0.922 [0.442–1.91]	ns (p=0.97)
Symptoms at 180-day visit	ICU, acute COVID-19, n = 32	No ICU, acute COVID-19, n = 113	145	1.24 [0.564–2.74]	ns (p=0.97)
Lung function impairment at 180-day visit	Male sex, n = 71	No male sex, n = 51	122	2.12 [0.964–4.85]	ns (p=0.1)
Lung function impairment at 180-day visit	Obesity, n = 22	No obesity, n = 100	122	1.94 [0.746–5]	ns (p=0.22)
Lung function impairment at 180-day visit	Current smoker, n = 3	No current smoker, n = 119	122	4.26 [0.397–93.4]	ns (p=0.3)
Lung function impairment at 180-day visit	Ex-smoker, n = 45	No ex-smoker, n = 77	122	1.95 [0.897–4.26]	ns (p=0.13)
Lung function impairment at 180-day visit	CVD, n = 49	No CVD, n = 73	122	1.57 [0.727–3.39]	ns (p=0.31)
Lung function impairment at 180-day visit	Hypertension, n = 35	No hypertension, n = 87	122	1.56 [0.683–3.54]	ns (p=0.34)
Lung function impairment at 180-day visit	PD, n = 23	No PD, n = 99	122	2.21 [0.869–5.62]	ns (p=0.13)
Lung function impairment at 180-day visit	COPD, n = 7	No COPD, n = 115	122	2.93 [0.615–15.5]	ns (p=0.23)
Lung function impairment at 180-day visit	Asthma, n = 9	No asthma, n = 113	122	1.03 [0.208–4.12]	ns (p=0.97)
Lung function impairment at 180-day visit	Metabolic disorders, n = 53	No metabolic disorders, n = 69	122	1.73 [0.807–3.73]	ns (p=0.22)
Lung function impairment at 180-day visit	Hypercholesterolemia, n = 24	No hypercholesterolemia, n = 98	122	1.03 [0.383–2.61]	ns (p=0.96)
Lung function impairment at 180-day visit	Diabetes, n = 21	No diabetes, n = 101	122	2.15 [0.816–5.64]	ns (p=0.16)
Lung function impairment at 180-day visit	CKD, n = 8	No CKD, n = 114	122	17.2 [2.9–328]	p=0.02
Lung function impairment at 180-day visit	GITD, n = 16	No GITD, n = 106	122	3.11 [1.07–9.42]	ns (p=0.061)
Lung function impairment at 180-day visit	Malignancy, n = 14	No malignancy, n = 108	122	4.47 [1.43–15.6]	p=0.025
Lung function impairment at 180-day visit	Immune deficiency, n = 6	No immune deficiency, n = 116	122	2.14 [0.38–12]	ns (p=0.43)
Lung function impairment at 180-day visit	Weight loss, acute COVID-19, n = 91	No weight loss, acute COVID-19, n = 31	122	1.56 [0.645–4.08]	ns (p=0.41)
Lung function impairment at 180-day visit	Dyspnea, acute COVID-19, n = 82	No dyspnea, acute COVID-19, n = 40	122	3.17 [1.31–8.58]	p=0.029
Lung function impairment at 180-day visit	Cough, acute COVID-19, n = 88	No cough, acute COVID-19, n = 34	122	0.856 [0.375–2.01]	ns (p=0.76)
Lung function impairment at 180-day visit	Fever, acute COVID-19, n = 92	No fever, acute COVID-19, n = 30	122	1.19 [0.496–3.01]	ns (p=0.76)
Lung function impairment at 180-day visit	Night sweat, acute COVID-19, n = 79	No night sweat, acute COVID-19, n = 43	122	0.39 [0.176–0.852]	p=0.033
Lung function impairment at 180-day visit	Pain, acute COVID-19, n = 65	No pain, acute COVID-19, n = 57	122	0.609 [0.282–1.3]	ns (p=0.26)
Lung function impairment at 180-day visit	GI symptoms, acute COVID-19, n = 46	No GI symptoms, acute COVID-19, n = 76	122	0.715 [0.316–1.57]	ns (p=0.47)
Lung function impairment at 180-day visit	Anosmia, acute COVID-19, n = 51	No anosmia, acute COVID-19, n = 71	122	0.895 [0.41–1.92]	ns (p=0.82)
Lung function impairment at 180-day visit	Impaired performance, acute COVID-19, n = 111	No impaired performance, acute COVID-19, n = 11	122	0.84 [0.238–3.38]	ns (p=0.82)
Lung function impairment at 180-day visit	Sleep disorders, acute COVID-19, n = 46	No sleep disorders, acute COVID-19, n = 75	121	0.7 [0.309–1.54]	ns (p=0.44)
Lung function impairment at 180-day visit	Anti-infectives, acute COVID-19, n = 63	No anti-infectives, acute COVID-19, n = 59	122	2.65 [1.22–6]	p=0.03
Lung function impairment at 180-day visit	Antiplatelet, acute COVID-19, n = 17	No antiplatelet, acute COVID-19, n = 105	122	4.8 [1.67–15.1]	p=0.011
Lung function impairment at 180-day visit	Anticoagulatives, acute COVID-19, n = 7	No anticoagulatives, acute COVID-19, n = 115	122	2.93 [0.615–15.5]	ns (p=0.23)
Lung function impairment at 180-day visit	Immunosuppression, acute COVID-19, n = 22	No immunosuppression, acute COVID-19, n = 100	122	2.45 [0.95–6.34]	ns (p=0.096)
Lung function impairment at 180-day visit	Anemia, 60-day visit, n = 11	No anemia, 60-day visit, n = 111	122	4.14 [1.17–16.7]	ns (p=0.053)
Lung function impairment at 180-day visit	Elevated ferritin, 60-day visit, n = 21	No elevated ferritin, 60-day visit, n = 100	121	1.37 [0.498–3.6]	ns (p=0.58)
Lung function impairment at 180-day visit	Elevated NTproBNP, 60-day visit, n = 44	No elevated NTproBNP, 60-day visit, n = 78	122	2.42 [1.11–5.33]	p=0.046
Lung function impairment at 180-day visit	Elevated D-dimer, 60-day visit, n = 50	No elevated D-dimer, 60-day visit, n = 72	122	3.23 [1.49–7.2]	p=0.0089
Lung function impairment at 180-day visit	Elevated CRP, 60-day visit, n = 17	No elevated CRP, 60-day visit, n = 105	122	6.6 [2.24–22.3]	p=0.0029
Lung function impairment at 180-day visit	Elevated IL-6, 60-day visit, n = 9	No elevated IL-6, 60-day visit, n = 113	122	20.2 [3.52–383]	p=0.013
Lung function impairment at 180-day visit	Iron deficiency, 60-day visit, n = 6	No iron deficiency, 60-day visit, n = 115	121	1.05 [0.142–5.65]	ns (p=0.96)
Lung function impairment at 180-day visit	Age over 65, n = 33	No age over 65, n = 89	122	2.55 [1.11–5.88]	p=0.046
Lung function impairment at 180-day visit	Hospitalized >7 days, acute COVID-19, n = 66	No hospitalized >7 days, acute COVID-19, n = 56	122	3.83 [1.7–9.21]	p=0.0045
Lung function impairment at 180-day visit	Any comorbidity, n = 93	No any comorbidity, n = 29	122	3.95 [1.39–14.2]	p=0.032
Lung function impairment at 180-day visit	>3 comorbidities, n = 41	No >3 comorbidities, n = 81	122	2.47 [1.12–5.48]	p=0.044
Lung function impairment at 180-day visit	Overweight or obesity, n = 72	No overweight or obesity, n = 50	122	1.24 [0.575–2.73]	ns (p=0.64)
Lung function impairment at 180-day visit	>6 symptoms, acute COVID-19, n = 34	No >6 symptoms, acute COVID-19, n = 88	122	0.538 [0.207–1.29]	ns (p=0.23)
Lung function impairment at 180-day visit	Persistent symptoms, 60-day visit, n = 96	No persistent symptoms, 60-day visit, n = 26	122	1.83 [0.702–5.39]	ns (p=0.3)
Lung function impairment at 180-day visit	Anti-S1/S2 IgG Q1, 60-day visit, n = 28	No anti-S1/S2 IgG Q1, 60-day visit, n = 84	112	0.245 [0.0675–0.704]	p=0.03
Lung function impairment at 180-day visit	Anti-S1/S2 IgG Q2, 60-day visit, n = 27	No anti-S1/S2 IgG Q2, 60-day visit, n = 85	112	2.23 [0.913–5.45]	ns (p=0.12)
Lung function impairment at 180-day visit	Anti-S1/S2 IgG Q3, 60-day visit, n = 28	No anti-S1/S2 IgG Q3, 60-day visit, n = 84	112	0.72 [0.27–1.78]	ns (p=0.55)
Lung function impairment at 180-day visit	Anti-S1/S2 IgG Q4, 60-day visit, n = 29	No anti-S1/S2 IgG Q4, 60-day visit, n = 83	112	1.88 [0.784–4.51]	ns (p=0.21)
Lung function impairment at 180-day visit	Ambulatory, acute COVID-19, n = 32	No ambulatory, acute COVID-19, n = 90	122	0.214 [0.0597–0.603]	p=0.017
Lung function impairment at 180-day visit	Hospitalized, acute COVID-19, n = 32	No hospitalized, acute COVID-19, n = 90	122	1.1 [0.458–2.56]	ns (p=0.85)
Lung function impairment at 180-day visit	Oxygen therapy, acute COVID-19, n = 32	No oxygen therapy, acute COVID-19, n = 90	122	1.33 [0.561–3.07]	ns (p=0.57)
Lung function impairment at 180-day visit	ICU, acute COVID-19, n = 26	No ICU, acute COVID-19, n = 96	122	2.56 [1.05–6.27]	ns (p=0.061)

CVD = cardiovascular disease; PD = pulmonary disease; COPD = chronic obstructive pulmonary disease; CKD = chronic kidney disease; GITD = gastrointestinal disease; GI = gastrointestinal; CRP = C-reactive protein; ICU = intensive care unit; CT = computed tomography.

**Appendix 1—table 3.** Feature cluster assignment scheme.

Cluster #	Variable
1	Male sex, CVD, hypertension, metabolic disorders, anti-infectives, acute COVID-19, elevated NTproBNP, 60-day visit, elevated D-dimer, 60-day visit, hospitalized >7 days, acute COVID-19, >3 comorbidities, overweight
2	Obesity, current smoker, ex-smoker, PD, COPD, asthma, hypercholesterolemia, diabetes, CKD, GITD, malignancy, immune deficiency, GI symptoms, acute COVID-19, anosmia, acute COVID-19, sleep disorders, acute COVID-19, antiplatelet, acute COVID-19, anticoagulatives, acute COVID-19, immunosuppression, acute COVID-19, anemia, 60-day visit, elevated ferritin, 60-day visit, elevated CRP, 60-day visit, elevated IL-6, 60-day visit, iron deficiency, 60-day visit, age over 65, > 6 symptoms, acute COVID-19, anti-S1/S2 IgG Q1, 60-day visit, anti-S1/S2 IgG Q2, 60-day visit, anti-S1/S2 IgG Q3, 60-day visit, anti-S1/S2 IgG Q4, 60-day visit, ambulatory, acute COVID-19, hospitalized, acute COVID-19, oxygen therapy, acute COVID-19, ICU, acute COVID-19, CT severity score 1–5 at 180-day visit, CT severity score >5 at 180-day visit, lung function impairment at 180-day visit
3	Weight loss, acute COVID-19, dyspnea, acute COVID-19, cough, acute COVID-19, fever, acute COVID-19, night sweat, acute COVID-19, pain, acute COVID-19, impaired performance, acute COVID-19, any comorbidity, persistent symptoms, 60-day visit, symptoms at 180-day visit

CVD = cardiovascular disease; PD = pulmonary disease; COPD = chronic obstructive pulmonary disease; CKD = chronic kidney disease; GITD = gastrointestinal disease; GI = gastrointestinal; CRP = C-reactive protein; ICU = intensive care unit; CT = computed tomography.

**Appendix 1—table 4.** Development of machine learning models. Outcome: outcome variable at the 180-day follow-up visit

Outcome	Classifier type	Caret method	Description	Package	Optimal arguments
CT abnormalities at 180-day visit	model	C5.0	C5.0	C50	trials = 10, model = tree, winnow = FALSE
		rf	Random Forest	randomForest	mtry = 27
		svmRadial	Support Vector Machines with Radial Basis Function Kernel	kernlab	sigma = 0.0105, C = 0.5
		nnet	Neural Network	nnet	size = 1, decay = 0
		glmnet	Elastic-Net Regularized Generalized Linear Models	glmnet	alpha = 0.1, lambda = 0.000431
	ensemble	glmnet	Elastic-Net Regularized Generalized Linear Models	glmnet	alpha = 1, lambda = 0.0523
CT severity score >5 at 180-day visit	model	C5.0	C5.0	C50	trials = 1, model = rules, winnow = TRUE
		rf	Random Forest	randomForest	mtry = 52
		svmRadial	Support Vector Machines with Radial Basis Function Kernel	kernlab	sigma = 0.00979, C = 0.5
		nnet	Neural Network	nnet	size = 1, decay = 0.1
		glmnet	Elastic-Net Regularized Generalized Linear Models	glmnet	alpha = 0.1, lambda = 0.0419
	ensemble	glmnet	Elastic-Net Regularized Generalized Linear Models	glmnet	alpha = 0.1, lambda = 0.00379
Symptoms at 180-day visit	model	C5.0	C5.0	C50	trials = 1, model = tree, winnow = FALSE
		rf	Random Forest	randomForest	mtry = 27
		svmRadial	Support Vector Machines with Radial Basis Function Kernel	kernlab	sigma = 0.0109, C = 1
		nnet	Neural Network	nnet	size = 3, decay = 0.1
		glmnet	Elastic-Net Regularized Generalized Linear Models	glmnet	alpha = 0.1, lambda = 0.000247
	ensemble	glmnet	Elastic-Net Regularized Generalized Linear Models	glmnet	alpha = 0.1, lambda = 0.0167
Lung function impairment at 180-day visit	model	C5.0	C5.0	C50	trials = 1, model = rules, winnow = FALSE
		rf	Random Forest	randomForest	mtry = 52
		svmRadial	Support Vector Machines with Radial Basis Function Kernel	kernlab	sigma = 0.0108, C = 0.5
		nnet	Neural Network	nnet	size = 1, decay = 0.1
		glmnet	Elastic-Net Regularized Generalized Linear Models	glmnet	alpha = 0.55, lambda = 0.0341
	ensemble	glmnet	Elastic-Net Regularized Generalized Linear Models	glmnet	alpha = 0.55, lambda = 0.0387

**Appendix 1—table 5.** Performance of machine learning classifiers. Outcome: outcome variable at the 180-day follow-up visit; Method: Caret method, Accuracy: model accuracy with 95% confidence intervals, Kappa: model kappa statistic with 95% confidence intervals, AUC: area under the curve.

Outcome	Total N	Events N	Method	Data set	Accuracy	Kappa	AUC	Sensitivity	Specificity
CT abnormalities at 180-day visit	109	49	C5.0	CV	0.72 [0.36–1]	0.43 [-0.35–1]	0.78	0.69	0.74
CT abnormalities at 180-day visit	109	49	C5.0	Training	1	1	1	1	1
CT abnormalities at 180-day visit	109	49	ensemble	CV	0.78 [0.63–0.93]	0.55 [0.26–0.85]	0.81	0.75	0.8
CT abnormalities at 180-day visit	109	49	ensemble	Training	0.93	0.85	0.98	0.86	0.98
CT abnormalities at 180-day visit	109	49	glmnet	CV	0.71 [0.3–1]	0.42 [-0.52–1]	0.79	0.71	0.72
CT abnormalities at 180-day visit	109	49	glmnet	Training	1	1	1	1	1
CT abnormalities at 180-day visit	109	49	nnet	cCV	0.67 [0.26–1]	0.35 [-0.38–1]	0.69	0.71	0.64
CT abnormalities at 180-day visit	109	49	nnet	Training	0.76	0.54	0.78	1	0.57
CT abnormalities at 180-day visit	109	49	rf	CV	0.73 [0.4–1]	0.45 [-0.33–1]	0.78	0.72	0.74
CT abnormalities at 180-day visit	109	49	rf	Training	1	1	1	1	1
CT abnormalities at 180-day visit	109	49	svmRadial	CV	0.75 [0.4–1]	0.51 [-0.25–1]	0.8	0.78	0.73
CT abnormalities at 180-day visit	109	49	svmRadial	Training	0.85	0.7	0.93	0.84	0.87
CT severity score >5 at 180-day visit	109	21	C5.0	CV	0.86 [0.67–1]	0.37 [-0.2–1]	0.7	0.39	0.98
CT severity score >5 at 180-day visit	109	21	C5.0	Training	0.87	0.5	0.7	0.43	0.98
CT severity score >5 at 180-day visit	109	21	ensemble	CV	0.88 [0.81–0.96]	0.51 [0.044–0.89]	0.75	0.45	0.98
CT severity score >5 at 180-day visit	109	21	ensemble	Training	0.89	0.57	0.65	0.48	0.99
CT severity score >5 at 180-day visit	109	21	glmnet	CV	0.84 [0.6–1]	0.34 [-0.25–1]	0.76	0.41	0.94
CT severity score >5 at 180-day visit	109	21	glmnet	Training	0.94	0.8	0.97	0.71	1
CT severity score >5 at 180-day visit	109	21	nnet	CV	0.79 [0.5–1]	0.31 [-0.29–1]	0.72	0.47	0.87
CT severity score >5 at 180-day visit	109	21	nnet	Training	0.99	0.97	1	0.95	1
CT severity score >5 at 180-day visit	109	21	rf	CV	0.84 [0.6–1]	0.34 [-0.25–1]	0.73	0.4	0.95
CT severity score >5 at 180-day visit	109	21	rf	Training	1	1	1	1	1
CT severity score >5 at 180-day visit	109	21	svmRadial	CV	0.87 [0.63–1]	0.43 [-0.23–1]	0.75	0.48	0.97
CT severity score >5 at 180-day visit	109	21	svmRadial	Training	0.92	0.68	0.99	0.57	1
Lung function impairment at 180-day visit	111	38	C5.0	CV	0.73 [0.33–1]	0.39 [-0.5–1]	0.7	0.54	0.84
Lung function impairment at 180-day visit	111	38	C5.0	Training	0.86	0.7	0.85	0.79	0.9
Lung function impairment at 180-day visit	111	38	ensemble	CV	0.75 [0.61–0.86]	0.39 [0.052–0.67]	0.72	0.48	0.89
Lung function impairment at 180-day visit	111	38	ensemble	Training	0.89	0.75	0.98	0.79	0.95
Lung function impairment at 180-day visit	111	38	glmnet	CV	0.74 [0.4–1]	0.37 [-0.36–1]	0.66	0.51	0.86
Lung function impairment at 180-day visit	111	38	glmnet	Training	0.83	0.59	0.89	0.61	0.95
Lung function impairment at 180-day visit	111	38	nnet	CV	0.65 [0.2–1]	0.2 [-0.5–1]	0.59	0.44	0.76
Lung function impairment at 180-day visit	111	38	nnet	Training	0.93	0.83	0.82	0.79	1
Lung function impairment at 180-day visit	111	38	rf	CV	0.73 [0.4–1]	0.35 [-0.33–1]	0.72	0.49	0.85
Lung function impairment at 180-day visit	111	38	rf	Training	1	1	1	1	1
Lung function impairment at 180-day visit	111	38	svmRadial	CV	0.72 [0.36–1]	0.35 [-0.44–1]	0.69	0.5	0.84
Lung function impairment at 180-day visit	111	38	svmRadial	Training	0.87	0.71	0.94	0.71	0.96
Symptoms at 180-day visit	133	65	C5.0	CV	0.6 [0.22–0.93]	0.2 [-0.51–0.87]	0.57	0.61	0.58
Symptoms at 180-day visit	133	65	C5.0	Training	0.93	0.86	0.96	0.89	0.97
Symptoms at 180-day visit	133	65	ensemble	CV	0.58 [0.41–0.74]	0.16 [-0.19–0.49]	0.6	0.52	0.63
Symptoms at 180-day visit	133	65	ensemble	Training	0.99	0.98	1	0.98	1
Symptoms at 180-day visit	133	65	glmnet	CV	0.56 [0.17–0.86]	0.13 [-0.64–0.72]	0.56	0.54	0.58
Symptoms at 180-day visit	133	65	glmnet	Training	0.85	0.7	0.92	0.82	0.88
Symptoms at 180-day visit	133	65	nnet	CV	0.59 [0.29–0.86]	0.17 [-0.52–0.72]	0.58	0.6	0.57
Symptoms at 180-day visit	133	65	nnet	Training	1	1	1	1	1
Symptoms at 180-day visit	133	65	rf	CV	0.56 [0.29–0.86]	0.13 [-0.46–0.71]	0.59	0.56	0.56
Symptoms at 180-day visit	133	65	rf	Training	1	1	1	1	1
Symptoms at 180-day visit	133	65	svmRadial	CV	0.54 [0.17–0.83]	0.089 [-0.67–0.67]	0.55	0.45	0.62
Symptoms at 180-day visit	133	65	svmRadial	Training	0.86	0.73	0.94	0.85	0.88

AUC = area under the curve; CT = computed tomography; glmnet = elastic-net regularized generalized linear models; nnet = neural networks; svmRadial = support vector machines with radial basis function kernel; rf = random forest; ensemble = model ensemble with elastic-net regularized generalized linear models.

**Appendix 1—table 6.** Performance of machine learning classifiers in the acute COVID-19 severity strata. Outcome: outcome variable at the 180-day follow-up visit; cohort subset: cohort acute COVID-19 severity strata (mild–moderate: outpatient or hospitalized without oxygen; severe–critical: oxygen therapy or ICU),

Outcome	Cohort subset	Total N	Events N	Method	Data set	AUC	Sensitivity	Specificity
CT abnormalities at 180-day visit	Whole cohort	109	49	C5.0	Training	1	1	1
CT abnormalities at 180-day visit	Mild–moderate COVID-19	58	18	C5.0	Training	1	1	1
CT abnormalities at 180-day visit	Severe–critical COVID-19	51	31	C5.0	Training	1	1	1
CT abnormalities at 180-day visit	Whole cohort	109	49	rf	Training	1	1	1
CT abnormalities at 180-day visit	Mild–moderate COVID-19	58	18	rf	Training	1	1	1
CT abnormalities at 180-day visit	Severe–critical COVID-19	51	31	rf	Training	1	1	1
CT abnormalities at 180-day visit	Whole cohort	109	49	svmRadial	Training	0.93	0.84	0.87
CT abnormalities at 180-day visit	Mild–moderate COVID-19	58	18	svmRadial	Training	0.9	0.61	0.95
CT abnormalities at 180-day visit	Severe–critical COVID-19	51	31	svmRadial	Training	0.96	0.97	0.7
CT abnormalities at 180-day visit	Whole cohort	109	49	nnet	Training	0.78	1	0.57
CT abnormalities at 180-day visit	Mild–moderate COVID-19	58	18	nnet	Training	0.92	1	0.85
CT abnormalities at 180-day visit	Severe–critical COVID-19	51	31	nnet	Training	0.5	1	0
CT abnormalities at 180-day visit	Whole cohort	109	49	glmnet	Training	1	1	1
CT abnormalities at 180-day visit	Mild–moderate COVID-19	58	18	glmnet	Training	1	1	1
CT abnormalities at 180-day visit	Severe–critical COVID-19	51	31	glmnet	Training	1	1	1
CT abnormalities at 180-day visit	Whole cohort	109	49	ensemble	Training	0.98	0.86	0.98
CT abnormalities at 180-day visit	Mild–moderate COVID-19	58	18	ensemble	Training	0.98	0.61	1
CT abnormalities at 180-day visit	Severe–critical COVID-19	51	31	ensemble	Training	1	1	0.95
CT severity score >5 at 180-day visit	Whole cohort	109	21	C5.0	Training	0.7	0.43	0.98
CT severity score >5 at 180-day visit	Mild–moderate COVID-19	58	6	C5.0	Training	0.57	0.17	0.98
CT severity score >5 at 180-day visit	Severe–critical COVID-19	51	15	C5.0	Training	0.75	0.53	0.97
CT severity score >5 at 180-day visit	Whole cohort	109	21	rf	Training	1	1	1
CT severity score >5 at 180-day visit	Mild–moderate COVID-19	58	6	rf	Training	1	1	1
CT severity score >5 at 180-day visit	Severe–critical COVID-19	51	15	rf	Training	1	1	1
CT severity score >5 at 180-day visit	Whole cohort	109	21	svmRadial	Training	0.99	0.57	1
CT severity score >5 at 180-day visit	Mild–moderate COVID-19	58	6	svmRadial	Training	0.98	0.17	1
CT severity score >5 at 180-day visit	Severe–critical COVID-19	51	15	svmRadial	Training	1	0.73	1
CT severity score >5 at 180-day visit	Whole cohort	109	21	nnet	Training	1	0.95	1
CT severity score >5 at 180-day visit	Mild–moderate COVID-19	58	6	nnet	Training	1	0.83	1
CT severity score >5 at 180-day visit	Severe–critical COVID-19	51	15	nnet	Training	1	1	1
CT severity score >5 at 180-day visit	Whole cohort	109	21	glmnet	Training	0.97	0.71	1
CT severity score >5 at 180-day visit	Mild–moderate COVID-19	58	6	glmnet	Training	0.94	0.33	1
CT severity score >5 at 180-day visit	Severe–critical COVID-19	51	15	glmnet	Training	1	0.87	1
CT severity score >5 at 180-day visit	Whole cohort	109	21	ensemble	Training	0.65	0.48	0.99
CT severity score >5 at 180-day visit	Mild–moderate COVID-19	58	6	ensemble	Training	0.38	0.17	0.98
CT severity score >5 at 180-day visit	Severe–critical COVID-19	51	15	ensemble	Training	0.74	0.6	1
Symptoms at 180-day visit	Whole cohort	133	65	C5.0	Training	0.96	0.89	0.97
Symptoms at 180-day visit	Mild–moderate COVID-19	64	30	C5.0	Training	0.97	0.9	1
Symptoms at 180-day visit	Severe–critical COVID-19	69	35	C5.0	Training	0.96	0.89	0.94
Symptoms at 180-day visit	Whole cohort	133	65	rf	Training	1	1	1
Symptoms at 180-day visit	Mild–moderate COVID-19	64	30	rf	Training	1	1	1
Symptoms at 180-day visit	Severe–critical COVID-19	69	35	rf	Training	1	1	1
Symptoms at 180-day visit	Whole cohort	133	65	svmRadial	Training	0.94	0.85	0.88
Symptoms at 180-day visit	Mild–moderate COVID-19	64	30	svmRadial	Training	0.93	0.77	0.85
Symptoms at 180-day visit	Severe–critical COVID-19	69	35	svmRadial	Training	0.95	0.91	0.91
Symptoms at 180-day visit	Whole cohort	133	65	nnet	Training	1	1	1
Symptoms at 180-day visit	Mild–moderate COVID-19	64	30	nnet	Training	1	1	1
Symptoms at 180-day visit	Severe–critical COVID-19	69	35	nnet	Training	1	1	1
Symptoms at 180-day visit	Whole cohort	133	65	glmnet	Training	0.92	0.82	0.88
Symptoms at 180-day visit	Mild–moderate COVID-19	64	30	glmnet	Training	0.91	0.73	0.88
Symptoms at 180-day visit	Severe–critical COVID-19	69	35	glmnet	Training	0.92	0.89	0.88
Symptoms at 180-day visit	Whole cohort	133	65	ensemble	Training	1	0.98	1
Symptoms at 180-day visit	Mild–moderate COVID-19	64	30	ensemble	Training	1	0.97	1
Symptoms at 180-day visit	Severe–critical COVID-19	69	35	ensemble	Training	1	1	1
Lung function impairment at 180-day visit	Whole cohort	111	38	C5.0	Training	0.85	0.79	0.9
Lung function impairment at 180-day visit	Mild–moderate COVID-19	55	14	C5.0	Training	0.81	0.71	0.9
Lung function impairment at 180-day visit	Severe–critical COVID-19	56	24	C5.0	Training	0.87	0.83	0.91
Lung function impairment at 180-day visit	Whole cohort	111	38	rf	Training	1	1	1
Lung function impairment at 180-day visit	Mild–moderate COVID-19	55	14	rf	Training	1	1	1
Lung function impairment at 180-day visit	Severe–critical COVID-19	56	24	rf	Training	1	1	1
Lung function impairment at 180-day visit	Whole cohort	111	38	svmRadial	Training	0.94	0.71	0.96
Lung function impairment at 180-day visit	Mild–moderate COVID-19	55	14	svmRadial	Training	0.88	0.5	0.98
Lung function impairment at 180-day visit	Severe–critical COVID-19	56	24	svmRadial	Training	0.98	0.83	0.94
Lung function impairment at 180-day visit	Whole cohort	111	38	nnet	Training	0.82	0.79	1
Lung function impairment at 180-day visit	Mild–moderate COVID-19	55	14	nnet	Training	0.7	0.64	1
Lung function impairment at 180-day visit	Severe–critical COVID-19	56	24	nnet	Training	0.89	0.88	1
Lung function impairment at 180-day visit	Whole cohort	111	38	glmnet	Training	0.89	0.61	0.95
Lung function impairment at 180-day visit	Mild–moderate COVID-19	55	14	glmnet	Training	0.84	0.29	0.95
Lung function impairment at 180-day visit	Severe–critical COVID-19	56	24	glmnet	Training	0.91	0.79	0.94
Lung function impairment at 180-day visit	Whole cohort	111	38	ensemble	Training	0.98	0.79	0.95
Lung function impairment at 180-day visit	Mild–moderate COVID-19	55	14	ensemble	Training	0.97	0.71	0.95
Lung function impairment at 180-day visit	Severe–critical COVID-19	56	24	ensemble	Training	0.98	0.83	0.94
CT abnormalities at 180-day visit	Whole cohort	109	49	C5.0	CV	0.78	0.69	0.74
CT abnormalities at 180-day visit	Mild–moderate COVID-19	58	18	C5.0	CV	0.69	0.43	0.8
CT abnormalities at 180-day visit	Severe–critical COVID-19	51	31	C5.0	CV	0.78	0.85	0.62
CT abnormalities at 180-day visit	Whole cohort	109	49	rf	CV	0.78	0.72	0.74
CT abnormalities at 180-day visit	Mild–moderate COVID-19	58	18	rf	CV	0.76	0.43	0.88
CT abnormalities at 180-day visit	Severe–critical COVID-19	51	31	rf	CV	0.71	0.88	0.47
CT abnormalities at 180-day visit	Whole cohort	109	49	svmRadial	CV	0.8	0.78	0.73
CT abnormalities at 180-day visit	Mild–moderate COVID-19	58	18	svmRadial	CV	0.75	0.56	0.9
CT abnormalities at 180-day visit	Severe–critical COVID-19	51	31	svmRadial	CV	0.76	0.92	0.4
CT abnormalities at 180-day visit	Whole cohort	109	49	nnet	CV	0.69	0.71	0.64
CT abnormalities at 180-day visit	Mild–moderate COVID-19	58	18	nnet	CV	0.67	0.59	0.77
CT abnormalities at 180-day visit	Severe–critical COVID-19	51	31	nnet	CV	0.62	0.78	0.39
CT abnormalities at 180-day visit	Whole cohort	109	49	glmnet	CV	0.79	0.71	0.72
CT abnormalities at 180-day visit	Mild–moderate COVID-19	58	18	glmnet	CV	0.78	0.66	0.78
CT abnormalities at 180-day visit	Severe–critical COVID-19	51	31	glmnet	CV	0.75	0.75	0.6
CT abnormalities at 180-day visit	Whole cohort	109	49	ensemble	CV	0.81	0.75	0.8
CT abnormalities at 180-day visit	Mild–moderate COVID-19	58	18	ensemble	CV	0.76	0.55	0.92
CT abnormalities at 180-day visit	Severe–critical COVID-19	51	31	ensemble	CV	0.79	0.87	0.55
CT severity score >5 at 180-day visit	Whole cohort	109	21	C5.0	CV	0.7	0.39	0.98
CT severity score >5 at 180-day visit	Mild–moderate COVID-19	58	6	C5.0	CV	0.55	0.13	0.98
CT severity score >5 at 180-day visit	Severe–critical COVID-19	51	15	C5.0	CV	0.76	0.49	0.97
CT severity score >5 at 180-day visit	Whole cohort	109	21	rf	CV	0.73	0.4	0.95
CT severity score >5 at 180-day visit	Mild–moderate COVID-19	58	6	rf	CV	0.58	0.033	0.96
CT severity score >5 at 180-day visit	Severe–critical COVID-19	51	15	rf	CV	0.76	0.55	0.93
CT severity score >5 at 180-day visit	Whole cohort	109	21	svmRadial	CV	0.75	0.48	0.97
CT severity score >5 at 180-day visit	Mild–moderate COVID-19	58	6	svmRadial	CV	0.59	0.13	0.97
CT severity score >5 at 180-day visit	Severe–critical COVID-19	51	15	svmRadial	CV	0.79	0.61	0.97
CT severity score >5 at 180-day visit	Whole cohort	109	21	nnet	CV	0.72	0.47	0.87
CT severity score >5 at 180-day visit	Mild–moderate COVID-19	58	6	nnet	CV	0.57	0.17	0.89
CT severity score >5 at 180-day visit	Severe–critical COVID-19	51	15	nnet	CV	0.76	0.59	0.84
CT severity score >5 at 180-day visit	Whole cohort	109	21	glmnet	CV	0.76	0.41	0.94
CT severity score >5 at 180-day visit	Mild–moderate COVID-19	58	6	glmnet	CV	0.63	0.17	0.97
CT severity score >5 at 180-day visit	Severe–critical COVID-19	51	15	glmnet	CV	0.78	0.51	0.89
CT severity score >5 at 180-day visit	Whole cohort	109	21	ensemble	CV	0.75	0.45	0.98
CT severity score >5 at 180-day visit	Mild–moderate COVID-19	58	6	ensemble	CV	0.64	0.17	0.98
CT severity score >5 at 180-day visit	Severe–critical COVID-19	51	15	ensemble	CV	0.78	0.57	0.99
Symptoms at 180-day visit	Whole cohort	133	65	C5.0	CV	0.57	0.61	0.58
Symptoms at 180-day visit	Mild–moderate COVID-19	64	30	C5.0	CV	0.58	0.62	0.56
Symptoms at 180-day visit	Severe–critical COVID-19	69	35	C5.0	CV	0.55	0.6	0.6
Symptoms at 180-day visit	Whole cohort	133	65	rf	CV	0.59	0.56	0.56
Symptoms at 180-day visit	Mild–moderate COVID-19	64	30	rf	CV	0.6	0.61	0.55
Symptoms at 180-day visit	Severe–critical COVID-19	69	35	rf	CV	0.57	0.52	0.58
Symptoms at 180-day visit	Whole cohort	133	65	svmRadial	CV	0.55	0.45	0.62
Symptoms at 180-day visit	Mild–moderate COVID-19	64	30	svmRadial	CV	0.54	0.48	0.59
Symptoms at 180-day visit	Severe–critical COVID-19	69	35	svmRadial	CV	0.56	0.43	0.66
Symptoms at 180-day visit	Whole cohort	133	65	nnet	CV	0.58	0.6	0.57
Symptoms at 180-day visit	Mild–moderate COVID-19	64	30	nnet	CV	0.58	0.63	0.54
Symptoms at 180-day visit	Severe–critical COVID-19	69	35	nnet	CV	0.58	0.58	0.6
Symptoms at 180-day visit	Whole cohort	133	65	glmnet	CV	0.56	0.54	0.58
Symptoms at 180-day visit	Mild–moderate COVID-19	64	30	glmnet	CV	0.56	0.57	0.6
Symptoms at 180-day visit	Severe–critical COVID-19	69	35	glmnet	CV	0.55	0.51	0.56
Symptoms at 180-day visit	Whole cohort	133	65	ensemble	CV	0.6	0.52	0.63
Symptoms at 180-day visit	Mild–moderate COVID-19	64	30	ensemble	CV	0.61	0.57	0.63
Symptoms at 180-day visit	Severe–critical COVID-19	69	35	ensemble	CV	0.6	0.49	0.63
Lung function impairment at 180-day visit	Whole cohort	111	38	C5.0	CV	0.7	0.54	0.84
Lung function impairment at 180-day visit	Mild–moderate COVID-19	55	14	C5.0	CV	0.61	0.37	0.86
Lung function impairment at 180-day visit	Severe–critical COVID-19	56	24	C5.0	CV	0.75	0.63	0.81
Lung function impairment at 180-day visit	Whole cohort	111	38	rf	CV	0.72	0.49	0.85
Lung function impairment at 180-day visit	Mild–moderate COVID-19	55	14	rf	CV	0.58	0.26	0.9
Lung function impairment at 180-day visit	Severe–critical COVID-19	56	24	rf	CV	0.79	0.62	0.79
Lung function impairment at 180-day visit	Whole cohort	111	38	svmRadial	CV	0.69	0.5	0.84
Lung function impairment at 180-day visit	Mild–moderate COVID-19	55	14	svmRadial	CV	0.56	0.29	0.88
Lung function impairment at 180-day visit	Severe–critical COVID-19	56	24	svmRadial	CV	0.75	0.62	0.79
Lung function impairment at 180-day visit	Whole cohort	111	38	nnet	CV	0.59	0.44	0.76
Lung function impairment at 180-day visit	Mild–moderate COVID-19	55	14	nnet	CV	0.47	0.29	0.83
Lung function impairment at 180-day visit	Severe–critical COVID-19	56	24	nnet	CV	0.62	0.52	0.67
Lung function impairment at 180-day visit	Whole cohort	111	38	glmnet	CV	0.66	0.51	0.86
Lung function impairment at 180-day visit	Mild–moderate COVID-19	55	14	glmnet	CV	0.55	0.21	0.88
Lung function impairment at 180-day visit	Severe–critical COVID-19	56	24	glmnet	CV	0.73	0.68	0.83
Lung function impairment at 180-day visit	Whole cohort	111	38	ensemble	CV	0.72	0.48	0.89
Lung function impairment at 180-day visit	Mild–moderate COVID-19	55	14	ensemble	CV	0.59	0.26	0.92
Lung function impairment at 180-day visit	Severe–critical COVID-19	56	24	ensemble	CV	0.78	0.61	0.85

AUC = area under the curve; CT = computed tomography; ICU = intensive care unit; glmnet = elastic-net regularized generalized linear models; nnet = neural network; svmRadial = support vector machines with radial basis function kernel; rf = random forest; ensemble = model ensemble with elastic-net regularized generalized linear models.
