# Supplementary material for: Investigating phenotypes of pulmonary COVID-19 recovery: A longitudinal observational prospective multicenter trial
Source: eLife. 2022 Feb 8;11:e72500. doi: 10.7554/eLife.72500 (PMC8896831; doi:10.7554/eLife.72500)
Supplement: Transparent reporting form [file elife-72500-transrepform1.docx]

***eLife’s* transparent reporting form**

We encourage authors to provide detailed information *within their submission* to facilitate the interpretation and replication of experiments. Authors can upload supporting documentation to indicate the use of appropriate reporting guidelines for health-related research (see [EQUATOR Network](http://www.equator-network.org/ \t _blank)), life science research (see the [BioSharing Information Resource](https://biosharing.org/)), or the [ARRIVE guidelines](http://www.plosbiology.org/article/info:doi/10.1371/journal.pbio.1000412) for reporting work involving animal research. Where applicable, authors should refer to any relevant reporting standards documents in this form.

If you have any questions, please consult our Journal Policies and/or contact us: [editorial@elifesciences.org](mailto:editorial@elifesciences.org).

**Sample-size estimation**

- You should state whether an appropriate sample size was computed when the study was being designed
- You should state the statistical method of sample size computation and any required assumptions
- If no explicit power analysis was used, you should describe how you decided what sample (replicate) size (number) to use

Please outline where this information can be found within the submission (e.g., sections or figure legends), or explain why this information doesn’t apply to your submission:

**Replicates**

- You should report how often each experiment was performed
- You should include a definition of biological versus technical replication
- The data obtained should be provided and sufficient information should be provided to indicate the number of independent biological and/or technical replicates

No explicit power analysis was done, the study participant number was limited by hospital admissions in the region and patient’s compliance. In the current report, a subset of the original CovILD cohort which completed the six-month follow-up visit was included (n = 145)

- If you encountered any outliers, you should describe how these were handled
- Criteria for exclusion/inclusion of data should be clearly stated
- High-throughput sequence data should be uploaded before submission, with a private link for reviewers provided (these are available from both GEO and ArrayExpress)

Please outline where this information can be found within the submission (e.g., sections or figure legends), or explain why this information doesn’t apply to your submission:

**Statistical reporting**

- Statistical analysis methods should be described and justified
- Raw data should be presented in figures whenever informative to do so (typically when N per group is less than 10)

This is a single cohort longitudinal study. Kinetic modeling was accomplished by mixed-effect logistic regression (random effect: individuals, fixed effect: time). Identification of risk factors was done with logistic regression. Clustering analysis was done with PAM or with a combined self-organizing map (SOM)/ hierarchical clustering algorithm. Multi-parameter modeling was done with machine learning (ML) classifiers. The stability of cluster assignment and ML performance was tested by 20-fold cross-validation.

- For each experiment, you should identify the statistical tests used, exact values of N, definitions of center, methods of multiple test correction, and dispersion and precision measures (e.g., mean, median, SD, SEM, confidence intervals; and, for the major substantive results, a measure of effect size (e.g., Pearson's r, Cohen's d)
- Report exact p-values wherever possible alongside the summary statistics and 95% confidence intervals. These should be reported for all key questions and not only when the p-value is less than 0.05.

Please outline where this information can be found within the submission (e.g., sections or figure legends), or explain why this information doesn’t apply to your submission:

(For large datasets, or papers with a very large number of statistical tests, you may upload a single table file with tests, Ns, etc., with reference to sections in the manuscript.)

**Group allocation**

- Indicate how samples were allocated into experimental groups (in the case of clinical studies, please specify allocation to treatment method); if randomization was used, please also state if restricted randomization was applied

The information on the statistical test used for the particular analysis can be found in Figure Legends. Except for the uni-variate logistic regression, p values are presented in the plot. P values and confidence intervals for the uni-variate risk modeling are provided in Appendix 1 – table 1.

- Indicate if masking was used during group allocation, data collection and/or data analysis

Please outline where this information can be found within the submission (e.g., sections or figure legends), or explain why this information doesn’t apply to your submission:

**Additional data files (“source data”)**

- We encourage you to upload relevant additional data files, such as numerical data that are represented as a graph in a figure, or as a summary table
- Where provided, these should be in the most useful format, and they can be uploaded as “Source data” files linked to a main figure or table
- Include model definition files including the full list of parameters used

As this is an observation longitudinal cohort study, no group allocation into study groups was done a priori. Patients were classified according to the acute disease severity as outpatients, hospitalized subjects without oxygen therapy, hospitalized subjects with oxygen therapy and ICU patients as described in Results/Patient characteristic.

- Include code used for data analysis (e.g., R, MatLab)
- Avoid stating that data files are “available upon request”

Please indicate the figures or tables for which source data files have been provided:

The full list of study parameters with their definitions is included in Appendix 1 - table 1. The complete R analysis pipeline and the anonymized study data in form of stratified study variables are available as a public GitHub repository: https://github.com/PiotrTymoszuk/CovILD_6_Months. The R code for the key tools used for uni-variate modeling and model quality control (Figures 4 and 5, <https://github.com/PiotrTymoszuk/lm_qc_tools>) as well as cluster analysis and its quality control (Figures 6 – 7, https://github.com/PiotrTymoszuk/clustering-tools-2) is available at GitHub.

Source data for Figures 2 – 10 are provided as a supplementary Excel file
